# Atrial fibrillation: Primary prevention, secondary prevention, and prevention of thromboembolic complications: Part 2

**DOI:** 10.3389/fcvm.2022.1060096

**Published:** 2023-03-10

**Authors:** Richard G. Trohman, Henry D. Huang, Parikshit S. Sharma

**Affiliations:** Section of Electrophysiology, Division of Cardiology, Department of Internal Medicine, Rush University Medical Center, Chicago, IL, United States

**Keywords:** pathophysiology, epidemiology, diet, lifestyle modification, pharmacological interventions, catheter ablation, surgical/hybrid ablation, thromboembolic prevention

## Abstract

Atrial fibrillation (AF), the most common sustained cardiac arrhythmia, once thought to be benign as long as the ventricular rate was controlled, is associated with significant cardiac morbidity and mortality. Increasing life expectancy driven by improved health care and decreased fertility rates has, in most of the world, resulted in the population aged ≥65 years growing more rapidly than the overall population. As the population ages, projections suggest that the burden of AF may increase more than 60% by 2050. Although considerable progress has been made in the treatment and management of AF, primary prevention, secondary prevention, and prevention of thromboembolic complications remain a work in progress. This narrative review was facilitated by a search of MEDLINE to identify peer-reviewed clinical trials, randomized controlled trials, meta-analyses, and other clinically relevant studies. The search was limited to English-language reports published between 1950 and 2021. *Atrial fibrillation was searched using the terms primary prevention, hyperthyroidism, Wolff-Parkinson-White syndrome, catheter ablation, surgical ablation, hybrid ablation, stroke prevention, anticoagulation, left atrial occlusion and atrial excision*. Google and Google scholar as well as bibliographies of identified articles were reviewed for additional references. In these two manuscripts, we discuss the current strategies available to prevent AF, then compare non-invasive and invasive treatment strategies to diminish AF recurrence. In addition, we examine the pharmacological, percutaneous device and surgical approaches to prevent stroke as well as other types of thromboembolic events.

## Introduction

Atrial fibrillation (AF) is the most common sustained tachyarrhythmia affecting millions of people worldwide. AF prevalence increases with advancing age. Left untreated, AF increases the risk and severity of heart failure, stroke and death (1). The mortality rate from AF as the primary or contributing etiology has been rising for over two decades (2).

In the United States, AF is the primary diagnosis in over 450,000 hospitalizations each year (3). AF contributes to more than 150,000 deaths each year (2). In 2019, AF was noted on 183,321 death certificates and was the underlying cause of death in 26,535 (2).

Worldwide AF is more common in developed nations and among individuals of European descent. Nevertheless, AF is hardly confined to these regions and demographics. In the Real-life global survey evaluating patients with atrial fibrillation (RealiseAF) a large-scale, cross-sectional international survey (from 26 countries) of 9,816 patients with AF who had ≥1 episode in the past 12 months, as AF progressed from paroxysmal to persistent and permanent types, the prevalence of comorbidities, including heart failure (32.9, 44.3, and 55.6%), coronary artery disease (30.0, 32.9, and 34.3%), cerebrovascular disease (11.7, 10.8, and 17.6%), and valvular disease (16.7, 21.2, and 35.8%), increased (4). Even the greater burden of morbidity and mortality experienced by young Indigenous Australians may, in part contributed to AF and it has speculated been that better prevention and management strategies may reduce this burden (5).

In part 2 we discuss catheter ablation in heart failure, compare and contrast catheter ablation modalities (including new energy sources), discuss surgical and hybrid AF ablative options, and review the pharnacological, interventional, and surgical options to prevent stroke and thromboembolism.

## AF catheter ablation in heart failure

A commonly adopted concept is that heart failure (HF) begets AF and AF begets HF due to shared pathophysiological mechanisms and risk factors (6). Several relatively small studies have compared PVI to pharmacologic rate control in patients with AF and heart failure (HF). In three studies, PVI demonstrated superior outcomes (7–9).

A multicenter randomized trial revealed that catheter ablation of AF was superior to amiodarone in achieving long-term freedom from AF as well as reducing unplanned hospitalization and mortality in patients with HF and persistent AF (10). MacDonald et al. randomized 41 patients with severe left ventricular systolic dysfunction [left ventricular ejection fraction (LVEF): 20.0 ± 5.5%] to receive catheter ablation or medical therapy and found no significant improvement in LVEF. A reduced ablation success rate (50%) in their advanced heart failure population may account for these findings (10).

The Pulmonary vein antrum isolation vs. AV node ablation with Bi-ventricular pacing for treatment of AF in patients with Congestive Heart Failure (PABA-CHF), compared AV junction ablation (the definitive approach to rate control) to PVI ± additional linear lesions and/or ablation of complex fractionated electrograms (11). Patients with symptomatic drug-resistant atrial fibrillation, a left ventricular ejection fraction (LVEF) ≤ 40%, and New York Heart Association (NYHA) class II or III HF were randomly assigned in a 1:1 fashion to undergo either pulmonary vein isolation or atrioventricular node (AVN) ablation with biventricular pacing.

In this randomized controlled trial, 41 patients underwent pulmonary vein isolation, and 40 underwent atrioventricular node ablation with biventricular pacing (via CRT-D). The primary end point was a composite of ejection fraction, distance on the 6-minute walk test, and Minnesota Living with Heart Failure (MLWHF) questionnaire score (11). Both groups had improved (lower) MLWHF scores after 6 months follow up, however the improvement was greater in the PVI group with a reduction from 89 ± 12 at baseline to 60 ± 8 vs. 89 ± 11 at baseline to 82 ± 14 in the AVN ablation group (P < 0.001).

In addition, the PVI group walked significantly farther (340 ± 49 m vs. 297 ± 36 m, P < 0.001), and had a significantly higher mean ejection fraction (35 ± 9% vs. 28 ± 6%, P < 0.001) (12). Arrhythmic progression, defined as AF that was becoming more advanced (e.g., from paroxysmal to persistent), did not occur in the patients who underwent pulmonary vein isolation but was noted in 30% of patients who underwent AV nodal (AVN) ablation with biventricular pacing (P < 0.001). The authors concluded PVI was superior to AVN ablation with biventricular pacing in heart failure patients with drug refractory atrial fibrillation (11).

Nevertheless, data from the Ablate and Pace for Atrial Fibrillation—cardiac resynchronization therapy (APAF-CRT) trial suggests that AV node/junction ablation and CRT should not be entirely dismissed. Patients with severely symptomatic permanent AF and narrow (< 110 ms) QRS complexes and ≥1 HF hospitalization in the previous year were randomized in a 1:1 manner to Ablation + CRT or to pharmacological rate control. In the rate control arm, therapy was optimized to attain a resting heart rate of < 110 beats/minute. The mean patient age was 72 ± 10 years (12, 13).

Patients were followed for a median of 16 months in phase I of the trial. Death due to HF, or HF hospitalization or worsening HF occurred in 20% of patients in the Ablation + CRT group and 38% of patients in the pharmacological rate control group (P = 0.013). Fewer patients in the Ablation + CRT group died from any cause or underwent hospitalization for HF (P = 0.008). Likewise, Ablation + CRT patients had a 36% decrease in symptoms and physical limitations related to AF at 1 year follow-up compared with the pharmacological rate control group (P = 0.004) (12).

In phase II of the ongoing APAF-CRT trial 133 patients were similarly randomized. The primary endpoint was time to all-cause mortality. The secondary endpoint was time to the composite of all-cause mortality or HF hospitalization, whichever occurred first. Both the primary and secondary endpoints were reduced significantly in the Ablation + CRT group compared to the pharmacological rate control group. The mortality benefit was not significantly different between patients with an EF ≤ 35% compared to those whose EF was >35% (13).

CASTLE-AF was a multicenter, randomized, controlled trial conducted to assess whether catheter ablation reduced morbidity and mortality compared with medical therapy (rate or rhythm control) in patients with paroxysmal or persistent AF as well as medically managed New York Heart Association (NYHA) class II–IV heart failure with an LVEF ≤ 35% (14). All patients were required to have had an ICD or CRT-D device with automatic daily remote-monitoring capabilities.

Participants in the trial included 179 patients randomized to ablation and 184 randomized to medical therapy. In patients randomized to medical therapy, efforts to maintain sinus rhythm were recommended, however, a rhythm control strategy was employed in only ~30%. For patients treated with rate control, ventricular rates of 60–80 beats per minute at rest and 90–115 beats per minute during moderate exercise were targeted. Twenty-eight patients in the ablation group crossed over to medical therapy and 18 patients in the medical therapy group crossed over to ablation (15).

The study's primary end point was a composite of death from any cause or worsening heart failure that led to an unplanned overnight hospitalization. Major secondary end points were death from any cause, death from cardiovascular disease, unplanned cardiovascular disease or heart failure hospitalization, any hospitalization, and cerebrovascular accident. In the ablation group, procedure-related adverse events and AF-free intervals were also assessed (14).

The primary composite end point was significantly lower in the ablation group. Likewise, death, cardiovascular death and hospitalizations were all significantly lower in the ablation group. AF recurred in 50% of ablation patients followed for 60 months, however, 63.1% of ablated patients were in sinus rhythm at their 60-month follow-up visit vs. 21.7% in the medical therapy group (P < 0.001) (14). A small, but statistically significant increase in LVEF was noted in the ablation group compared to the medical therapy group. The median absolute increase in LVEF from baseline to the 60-month follow-up visit was 8.0% in the ablation group and was 0.2% in the medical-therapy group (P = 0.005). The authors concluded that catheter ablation was associated with lower mortality rates from any cause, reduced rates of AF hospitalization, a lower AF burden and improved LVEF (14).

CABANA, an international, multicenter trial, randomized 2,204 AF patients to a strategy of catheter ablation or drug therapy, and followed them longitudinally for an average of 4 years for mortality and a comprehensive series of clinically relevant cardiovascular outcomes, quality of life, and AF recurrence (15, 16). Patients randomized to ablation had a significantly lower AF recurrence rate (49.9 vs. 69.5%, P < 0.001), but did not experience a significant change in the primary composite end point of disabling stroke, serious bleeding, cardiac arrest or death compared with patients randomized to receive drug therapy (8.0 vs. 9.2%, P = 0.30) (15, 16). The secondary end point of death and cardiovascular hospitalization, was significantly lower in the ablation group (51.7 vs. 58.1%, P = 0.001), primarily due to a reduced incidence of hospitalizations for antiarrhythmic drug titration, toxicity, and pacemaker implantation (15, 16). Using three prespecified QOL assessments, both treatment groups had significant improvements in quality of life, however, the improvement was significantly greater in the catheter ablation group (P < 0.001 for all 3 assessments) (16, 17). No clinically relevant treatment-related differences between men and women in the primary and secondary clinical outcomes of CABANA were noted, and there were no gender differences in adverse events (18). In the North American CABANA cohort, catheter ablation significantly improved major clinical outcomes in racial/ethnic minorities compared with drug therapy. Although primary event rates in racial/ethnic minority and non-minority participants were similar in the ablation arm, racial and ethnic minorities had a much higher event rate than non-minority participants when randomized to drug therapy (19).

In AF patients with clinically stable HF at CABANA trial entry, catheter ablation produced clinically important improvements in freedom from AF recurrence, quality of life and survival relative to drug therapy (20). Unfortunately, only 9.3% of patients with available data had an EF < 40% and in 11.7% EF ranged between 40 and 50% (20). In an editorial accompanying this report, Rosenfeld and Enriquez noted that there were too few patients with HF and reduced ejection fraction (HFrEF) to draw conclusions about this subgroup. They suggested that this was the largest randomized trial to compare AF catheter ablation to drug therapy in patients with HF and preserved ejection fraction (HFpEF) (21).

## Ablation modalities: Comparative efficacy and new techniques

The two ablation technologies most frequently used for pulmonary vein isolation differ in their energy source and mode of application. Radiofrequency (RF) current has been delivered in a point-by-point fashion, leading to tissue heating and cellular necrosis. The cryoballoon was developed create circular lesions around each pulmonary vein in a comparatively simpler manner. Cryogenic energy delivered via the balloon in a single-step (AKA “single shot”) mode, leads to necrosis by freezing.

The FIRE AND ICE Investigators were the first to compare RF and cryoablation for drug-refractory paroxysmal AF. Cryoballoon ablation efficacy was non-inferior to radiofrequency ablation for treatment of drug-refractory paroxysmal AF. There was no significant difference between the two methods in overall safety. RF ablation for AF requires limited fluoroscopy use because catheter guidance is achieved via electroanatomical mapping. However, cryoballoon ablation requires more extensive fluoroscopic guidance to position the balloon catheter at the pulmonary venous antra (22).

A 2020 meta-analysis comparing saline-irrigated radiofrequency ablation with contact force measurement (SRFA-CF) to the 2nd generation cryoballoon ablation (CBA-2G) revealed no difference in freedom from atrial arrhythmia, acute PV isolation, and total complications (23).

A retrospective comparison between a novel cryoballoon technology (POLARx; Boston Scientific Corporation, Marlborough, MA, USA) and the Arctic Front PRO (Medtronic Inc., Minneapolis, MN, USA) revealed similar efficacy, however the newer POLARx procedure times were 1.5 times longer and required more cryoablation applications (24, 25).

The fourth-generation Medtronic cryoballoon (Arctic Front Advance Pro; CB4) was retrospectively compared to their second generation cryoballoon (Arctic Front Advance; CB2). The Arctic Front Advance PRO was designed with a shorter tip (8 mm) in order to allow more proximal mapping catheter placement in the pulmonary vein and increase visualization of real-time pulmonary vein recordings during ablation. Real-time pulmonary vein isolation was visualized in 33.3% of the veins in CB2 group and 74.7% of the veins in the CB4 group (P < 0.001). This facilitated observation of the time-to-effect (TTE), which for practical purposes, is the time from the onset of the freeze until the pulmonary vein is isolated. CB4 significantly reduced procedure and fluoroscopy times, as well as the number of cryoapplications (26).

In 2021, the Arctic Front Advance and Arctic Front Advance Pro Cardiac Cryoablation Catheters were approved by the U.S. Food and Drug Administration for treatment of drug refractory recurrent symptomatic paroxysmal and persistent AF as well as an initial rhythm control strategy for treatment of recurrent symptomatic paroxysmal and persistent AF. The Freezor MAX Cardiac Cryoablation Catheter was also approved as an adjunctive device to complete pulmonary vein isolation by closing gaps, for focal triggers, and to creation an ablation line between the tricuspid valve and the inferior vena cava (27).

The endoscopic laser balloon ablation system (Heartlight^®^, CardioFocus, Inc., Marlborough, MA, USA), currently in its third generation, is the only commercially available device utilizing laser technology for pulmonary vein antral isolation. A multicenter, non-inferiority randomized control trial including 353 drug-refractory paroxysmal AF patients compared laser balloon ablation (LBA) to RF ablation for paroxysmal AF treatment. Freedom from recurrent symptomatic AF at 12 months was similar in both groups. Complication rates were non-inferior in the LBA group compared to the RF group, with pulmonary vein stenosis being less common in the LBA (28, 29).

In a multicenter European trial involving 135 persistent AF patients, comparable clinical efficacy of LBA and RF ablation was seen for the primary endpoint of freedom from AF between 90 and 365 days (median duration 14 months) in both treatment groups (71.2% in the LBA, vs. 69.3% in the RF group, P = 0.40). Similar complication rates were noted in both groups (28, 30).

While there are fewer head-to-head comparisons between cryoballoon ablation (CBA) and LBA techniques, the limited data available suggests comparable, if not slightly greater, procedural efficacy of LBA over cryoballoon ablation (28, 31, 32). CBA and LBA techniques have been found to have similar safety profiles (28).

In Part 1 of this series, we discussed the possible benefits of left atrial posterior wall ablation for persistent AF. In contrast, Verma et al., found no reduction in the AF recurrence rate when either linear ablation or ablation of complex fractionated electrograms was added to pulmonary vein isolation in patients with persistent AF (33). Other targets have been chosen for ablation of persistent AF (rotors, voltage-guided ablation, ganglionic plexus ablation), and it remains unclear if targeting anything beyond isolating the pulmonary veins is universally applicable, or of incremental benefit (33–35).

Ablation index (AI; Biosense Webster, Inc., Diamond Bar, CA, USA) is a commercially available new marker/index that combines contact force, radiofrequency time, and radiofrequency power in a non-linear formula in real time. In the PRAISE Study, 40 consecutive patients with persistent AF of < 12 months duration and no significant structural heart disease underwent AI-guided pulmonary vein isolation via point-by-point wide area circumferential ablation. Pre-specified AI targets were used in different regions. Ablation index regional target-guided ablation resulted in 93% of pulmonary veins remaining durably isolated at repeat (2 months) electrophysiology study. AI-guided PVI only ablation strategy was associated with a successful outcome in the vast majority of patients for >12 months, most likely due to durable PVI (35).

In a meta-analysis of 11 studies, including 2,306 patients, 1,046 (45.4%) underwent AI-guided ablation and 1,260 (54.5%) underwent non-AI guided ablation (control group). AI-guided catheter ablation was associated with significantly reduced procedural, fluoroscopy, and ablation times. PV isolation after single encirclement was more frequent in the AI-guided radiofrequency group, while acute PV reconnection was more frequently observed in the non-AI-guided ablation group. Data available data from 6 studies revealed that AI-guided catheter ablation was associated with significantly less AF recurrence compared to non-AI ablation studies (P = 0.0003) (36).

Newer ablation technologies such as the use of phased array radiofrequency ablation employ specialized circular catheters to achieve PVI. The original multi-electrode phased array radiofrequency pulmonary vein ablation catheter (PVAC, Medtronic Inc., Minneapolis, MN, USA) was designed to allow circumferential pulmonary vein isolation using a “single-shot” approach with the aim of reducing procedure time, while retaining efficacy and safety. Concerns about asymptomatic cerebral emboli led to development of the second-generation multi-electrode catheter (PVAC GOLD, Medtronic Inc., Minneapolis, MN, USA) (37). The electrodes were changed from platinum to gold, the number of electrodes reduced to nine from ten with PVAC, the inter-electrode spacing was increased from 3.00 to 3.75 mm and a 20° forward tilt was added to the array (37).

A recently published large registry of 1,017 patients, reported that PVI was successfully achieved in 95–99% of paroxysmal, persistent and long-standing AF patients. Procedure times were reduced, and safety was maintained (38). Additional phased array products (Medtronic Inc., Minneapolis, MN, USA) include the Multi-Array Septal Catheter (MASC) and the Multi-Array Ablation Catheter (MAAC) which are designed to facilitate elimination of non-pulmonary vein triggers and arrhythmogenic substrates in the atria (38). Pending additional investigation none of these products have received approval from the U.S. Food and Drug Administration (FDA).

Pulsed-field ablation (PFA) is currently being investigated for use as an alternate non-thermal energy source for ablation of cardiac arrhythmias. PFA employs high voltage electrical fields applied in close proximity to targeted tissues to create injury via irreversible electroporation. Increased cellular permeability from pore formation in the plasma membrane may result in in homeostatic changes and cell death from apoptosis (39).

While tissue-catheter contact is pivotal in creation of adequate lesions during thermal ablation, PFA's effects on tissue are proximity dependent because they are a result of the electric field created. Electroporation depends on current conduction between two electrodes. In accordance with Ohm's law current is directly proportional to voltage and inversely proportional to tissue resistence. Typically when current is applied between electrodes, the local current density in the tissue decreases with the square of the distance from the electrodes (39).

PFA is uniquely tissue specific. Its effect on tissue is dependent on tissue characteristics, fiber orientation, and heterogeneities in the local environment. Myocardium is very susceptible to irreversible injury, however, the esophagus, phrenic nerves, pulmonary veins (stenosis does not appear to occur), and coronary arteries are relatively resistant to injury (39).

In 2019, Reddy et al. reported the results of the IMPULSE (a safety and feasibility study of the IOWA approach endocardial ablation system to treat atrial fibrillation) and PEFCAT (a safety and feasibility study of the FARAPULSE endocardial ablation system to treat paroxysmal atrial fibrillation) studies (40). These were two-center, first-in-human, non-randomized feasibility trials of PFA for paroxysmal AF ablation. These studies were funded by Farapulse, Inc. [Farapulse Menlo Park, California, USA (formerly Iowa Approach)] (40).

All pulmonary veins were acutely isolated by monophasic or biphasic PFA. Although the optimal waveform (for safety and efficacy) for catheter ablation utilizing PFA has not been completely determined (39, 40) after waveform refinement, as the investigators gained experience and the system went through iterations, the durability of PVI at 3 months improved from 18 to 100% of patients with all PVs isolated. Patients in the latter group were treated with biphasic pulses at 1800–2000 V, with eight or more pulse deliveries per catheter position, and multiple catheter positions for each vein. There was one procedure-related pericardial tamponade, but over a median follow-up of 120 days there were no additional primary adverse events such as phrenic nerve injury, PV stenosis, esophageal injury or stroke. The authors concluded that PFA preferentially affected myocardial tissue, allowing facile ultra-rapid PV isolation with excellent durability and chronic safety (40).

In 2021, these authors and additional investigators reported the 1 year follow up results of IMPULSE, PEFCAT and PEFCAT II (expanded safety and feasibility study of the FARAPULSE endocardial multi ablation system to treat paroxysmal atrial fibrillation) (41). Acute PVI was achieved in 100% of 121 patients with PFA. Remapping performed at 93.0 ± 30.1 days revealed durable PVI in 96% of patients treated with optimized biphasic energy PFA. Primary adverse events occurred in 2.5% of patients (2 pericardial effusions or tamponade, 1 hematoma) and, in addition, there was 1 transient ischemic attack. There were no instances of atrioesophageal fistula, stroke, PV stenosis >70%, or phrenic nerve injury (41).

The median follow-up duration was 360 days and 80.2% of patients were followed for at least 1 year. The Kaplan-Meier estimate for freedom from atrial tachycardia, atrial flutter, and AF at 1 year was 78.5 ± 3.8%. The authors concluded that the data help allay concerns that the novel non-thermal PFA ablation modality masks undiscovered compromises to clinical success (41).

In 2021, Farapulse (Farapulse Menlo Park, California, USA) became the first company to commercialize a cardiac PFA technology after receiving CE Mark for the FARAPULSE PFA System in Europe in 2021. The company also initiated its pivotal U.S. IDE trial (the ADVENT trial) in the first quarter of 2021. In 6/21, Boston Scientific (Marlborough, MA, USA) announced it would acquire the FARAPULSE PFA System (42).

Medtronic, Inc. (Minneapolis, MN, USA) has initiated The PULSED AF trial, a prospective, non-randomized, multi-center clinical trial aiming to enroll up to 500 patients for treatment with their PulseSelect System in as many as 50 sites in the U.S., Canada, Europe, and Australia (43).

The initial in-human pilot phase evaluated the feasibility and efficacy of pulmonary vein isolation using a novel (PulseSelect; Medtronic, Inc., Minneapolis, MN, USA) PFA system delivering bipolar, biphasic electrical fields through a circular multielectrode array catheter (44).

Acute electrical isolation was achieved in 100% of pulmonary veins (*n* = 152) in 38 patients with paroxysmal or persistent AF. The total procedure time was 160 ± 91 mins, left atrial dwell time was 82 ± 35 mins, and fluoroscopy time was 28 ± 9 mins (44).

In the United States, Farapulse and Boston Scientific have launched the ADVENT trial (a prospective randomized pivotal trial of the FARAPULSE pulsed field ablation system compared with standard of care ablation in patients with paroxysmal atrial fibrillation) (45). The ADVENT trial is a randomized controlled trial that will involve at least 350 patients [estimated enrollment 900 patients (46)] in over 30 US centers. Patients will be randomized in a 1:1 fashion to either standard ablation (via radiofrequency or cryoballoon) or FARAPULSE PFA. The primary endpoint will be freedom from AF for 12 months after a single ablation procedure (46).

In 2007, Edgerton et al. introduced a minimally invasive surgical approach that combined pulmonary vein antral isolation and partial autonomic denervation. The investigators concluded that pulmonary vein isolation combined with targeted partial autonomic denervation was a safe and efficacious approach for the treatment of paroxysmal AF (47).

This concept has been extended to AF catheter ablation. The five major left atrial autonomic ganglionic plexi (GPs) locations are the superior left GP, inferior left GP, anterior right GP, inferior right GP, and within the ligament of Marshall. They can be located by recording highly fractionated atrial potentials (FAPs) which are also known as complex fractionated atrial electrograms (CFAEs). High frequency stimulation (HFS) of the left atrial endocardium delivered via a mapping or ablation catheter is useful to confirm a GP's location.

A positive (transient AV block or a decrease in mean ventricular rate > 50%) HFS response confirms the location. Unfortunately, the utility of catheter ablation of autonomic ganglia as an initial or repeat ablation strategy for paroxysmal, persistent, and long-standing persistent AF is not well established (48).

The work of Wolf, Edgerton, and others played a key role the development of hybrid ablation which was first described in 2012 (see below).

## A few words of caution

While there is great enthusiasm for catheter ablation of AF, the net clinical benefit depends on the safety of the ablation procedure in real-world practice (49). AF ablation is a complex procedure with a relatively high inherent risk (48). It should be no surprise that operator inexperience and low hospital procedure volumes are important predictors of complications. Most of the risk associated with catheter ablation of AF occurs during the acute procedural period (48). Even in highly experienced centers, about 4–5% of patients will experience complications (50). Major complications include thromboembolism (stroke and transient ischemic attacks), cardiac tamponade, significant pulmonary vein stenosis (≥70%), deep vein thrombosis, retroperitoneal bleeding and/or hematoma, femoral pseudoaneurysms and arteriovenous fistulae, atrioesophageal fistulae, and death (48, 50).

Vascular injuries are the most common complications of AF ablation. However, the incidence of serious complications such as arteriovenous fistula, femoral pseudoaneurysm, and retroperitoneal bleeding ranges from 0.2–1.5%. Cardiac tamponade (reported incidence 0–6%) is the most common life-threatening complication of AF ablation. Thromboembolic events (reported incidence 0–7%) usually occur within 24 h after ablation (the risk remains high for 2 weeks). In experienced hands, the incidence of symptomatic PV stenosis approaches zero. However, it is difficult to treat and can (rarely) result in death. Symptomatic patients may require pulmonary venous angioplasty and stenting. Atrioesophageal fistulae (incidence 0.2–0.11%), usually manifest 2–4 weeks post ablation. Treatment of atrioesophageal fistulae requires urgent surgical repair. Even with surgical repair, mortality is 34% (48). Esophageal injury has been observed most frequently with percutaneous radiofrequency ablation, but other energy sources (including but not limited to cryoablation) have been implicated as well. Luminal esophageal temperature (LET) monitoring does not prevent ablation-induced esophageal lesions (51). Likewise, mechanical techniques that aim to shift the esophagus away from the tip of the ablation catheter have not been proven to have a favorable risk-benefit ratio (52). Promising results have been noted with active esophageal cooling. In a large, randomized trial 188 patients undergoing RF ablation were randomized to active esophageal cooling vs. placement of a single-sensor temperature probe. Ultimately, 60 patients in each group underwent endoscopy 7 days post-ablation. Endoscopy demonstrated significantly fewer thermal injuries in patients protected by active esophageal cooling compared to the control group (2/60 vs. 12/60, P = 0.008) (53).

Phrenic nerve (PN) palsy is most commonly associated with cryoballoon ablation (incidence 3.5%−11.2%) but may occur after ablation with other energy sources. The right PN is most commonly affected during ablation in the right pulmonary veins or (less commonly) the superior vena cava. During cryothermal ablation, most PN injuries are transient, resolving within minutes. Persistent injuries are less common. Fortunately, cryothermal ablation is rarely associated with permanent PN paralysis. Even in persistent cases, nearly all recover within 12 months, however, 18–24 months may be required (48).

Asymptomatic cerebral emboli occur in 2–15% of procedures. Air embolism (usually due to suboptimal management of the transseptal sheath) occurs in < 1%. Coronary artery stenosis/occlusion occurs < 0.1% of AF catheter ablations and is addressed by prompt angioplasty. Pyloric spasm and gastric hypomotility has been reported to occur in as few as 0% and as many as 17% of procedures (48). Our experience suggests this is a very rare with an incidence of about 0.3% (54).

Radiation exposure has been associated with erythema, burns, hair loss, and necrosis of the skin as well as malignancies, cataracts, genetic diseases, and thyroid dysfunction.

Deterministic adverse effects occur after the radiation dose exceeds a specific threshold. Stochastic adverse effects are not threshold dependent. While their onset may increase in proportion to the intensity of exposure, their severity cannot be predicted by the radiation dose delivered (55).

Catheter ablation for cardiac arrhythmias has traditionally been guided by fluoroscopy. AF catheter ablation has required significantly longer fluoroscopy times and more radiation exposure than less complex catheter ablation procedures (48). Two recent meta-analyses have compared the use of low fluoroscopic (LF) and zero fluoroscopic approaches to conventional fluoroscopically guided AF ablation (56, 57). The low fluoroscopy technique had no significant differences in clinical efficacy or safety when compared to the conventional approach (56). In addition, important procedure parameters such as total procedure duration, fluoroscopy time, and dose area product were all significantly lower when employing an LF PVI approach (56). Zero fluoroscopy was also highly effective with acute procedure success rates and arrhythmia recurrence-free survival comparable to a more traditional conventional and low fluoroscopic approaches, but without radiation exposure to the patient, operator, and lab staff (57).

Recurrence of AF post ablation is observed in 30%−50% of patients by 1 year (58, 59). Recurrence is particularly problematic in patients with persistent AF. In addition, left atrial enlargement is generally acknowledged as a risk factor (60). AF catheter ablation can be an effective, durable long-term (≥3 years) therapeutic strategy for some patients however, multiple ablative procedures may be required (61).

The APPLE score [one point for age > 65 years, persistent AF, impaired eGFR (< 60 ml/min/1.73 m^2^), LA diameter ≥43 mm, EF < 50%] has been associated with AF recurrence and is a better predictor than the CHADS2 and CHA2DS2-VASc scores (60). Additional scores developed for predicting AF recurrence include the BASE-AF2 score, HATCH score, MB-LATER score ALARMc and CAAP-AF scores (62, 63).

The BASE-AF2 was derived to predict recurrences in AF patients after cryoballoon ablation. It seems to be best in patients with pulmonary disease (62, 63). The MB-LATER score [male, bundle branch block, left atrium ≥47 mm, type of AF (paroxysmal, persistent or long-standing persistent), and ER-AF = early recurrent AF], had better predictive ability for very late AF recurrence (>12 months) than the APPLE, ALARMEc, BASE-AF2, CHADS_2_, CHA_2_DS_2_VASc or HATCH scores (63). The ALARMEc score (nonparoxysmal AF [NPAF], normalized LA area >10.25, eGFR < 68 ml/min, metabolic syndrome and cardiomyopathy) has a good predictive ability of AF recurrence during a 2-year follow-up after redo procedure(s) (63). The CAAP-AF score was developed to predict AF freedom after RF ablation. CAD, LA diameter, age, persistent or long-standing persistent AF, antiarrhythmics failed and female sex are included in this predictive model (63).

Although favorable composite data exists (64), the long-term impact of AF catheter ablation on stroke (65) and mortality (64) has yet to be defined.

## Surgical AF ablation

The first Cox-maze (CMP) procedure was performed in 1987 (66–68). The original procedure was effective but caused two undesirable problems: (1) frequent inability to achieve appropriate sinus tachycardia in response to maximal exercise and (2) occasional dysfunction of the left atrium (67, 69).

In the maze II procedure, the incision through the sinus node area was eliminated. Additionally, the transverse atriotomy across the dome of the left atrium was moved posteriorly to permit better intra-atrial conduction. Unfortunately, this modification required complete transection of the superior vena cava to gain left atrial exposure (67, 70). The Cox-maze III procedure placed the septal incision posterior to the superior vena cava orifice and improved the exposure of the left atrium. The technically less demanding Cox-maze III resulted in a greater incidence of postoperative sinus rhythm, improved long-term sinus node function, decreased pacemaker requirements, less arrhythmia recurrence, and improved long-term atrial transport function (67, 71).

Cox-maze III results were compared in patients with lone AF who underwent “stand-alone AF surgery” and patients who underwent “concomitant AF surgery.” The most common concomitant surgeries were mitral repair, mitral valve replacement, and coronary artery bypass grafting. In the lone AF group 72/112 (64%) patients had paroxysmal AF and 40 (36%) had persistent AF. The mean follow-up duration was 5.4 ± 3.0 and 5.4 ± 2.7 years in the lone and concomitant groups respectively. At the conclusion of follow-up, 78 (79.6%) of stand-alone patients were not in AF and free of antiarrhythmic medications. Similarly, 58 (73.4%) patients in the concomitant group were in sinus rhythm and off all antiarrhythmic medication. An additional 19 (24%) patients were free of AF but were taking medications. There was no significant difference between the groups (72).

Minimally invasive approaches, as well the use of radiofrequency and cryothermal procedures (to replace the so-called “cut and sew” techniques) were introduced in 2002. Alternate energy sources such radiofrequency, microwave and laser were also tried. In general, alternate energy sources do not all function as well as radiofrequency or cryoablation (73). Microwave energy results were less than satisfactory (74, 75). When new ablation technology, including bipolar RF energy and new cryoablation systems, is used in the open chest and a complete biatrial Cox-maze lesion set performed, the procedure has been deemed the Cox-maze IV procedure. The Cox-maze IV procedure was introduced in 2002 (76). In this modification, the pulmonary veins were isolated bilaterally, and a connecting lesion was applied rather than performing the original box lesion around all four pulmonary veins. Importantly, cross-clamp times were shorter with the Cox-Maze IV procedure and a Cox-Maze IV procedure through a small, right inframammary incision was perfected. Two years after its original iteration, the final version of the CMP-IV isolated the entire posterior left atrium by adding a superior connecting line, to “recreate” the box lesion-set (73).

In 2007, Lall et al. compared 242 patients who underwent surgically based AF ablation using COX-maze III (154 patients) and COX-maze IV (88 patients) techniques. Their analysis revealed no significant difference in freedom from AF at 12 months (96% for COX-maze III and 93% for COX-MAZE IV group). The authors concluded that bipolar radiofrequency ablation simplified the Cox-Maze procedure, making it applicable to virtually all patients with atrial fibrillation undergoing concomitant cardiac surgery (77).

Data collected prospectively (during 1992–2010) from 212 consecutive patients (mean age, 53.5 ± 10.4 years; 78% male) who underwent a stand-alone Cox-maze-III (*n* = 112) or Cox-maze IV (*n* = 100) procedure was assessed. The median preoperative AF duration was six years. AF types were (paroxysmal 48%) and persistent or long-standing persistent (52%). Overall, 30-day mortality was 1.4%. There were no intraoperative deaths. A strict follow-up regimen was implemented with all patients having ECGs or 24-h Holter monitoring at 3, 6, and 12 months and annually thereafter. Follow-up, at a mean of 3.6 ± 3.1 years, revealed, freedom from AF was 93% (82% off antiarrhythmics). At 10 years, freedom from symptomatic AF was 85%. One late stroke occurred, with 80% of patients not receiving anticoagulation therapy. The less invasive CMP-IV had shorter cross-clamp times (41 ± 13 vs. 92 ± 26 mins; P < 0.001) while maintaining high success rates (90% freedom from AF; 84% freedom without antiarrhythmics at 2 years) (76).

In 2005, Wolf described video-assisted bilateral thoracoscopic pulmonary vein isolation with to plus excision of the left atrial appendage as feasible and safe. Although 91.3% of patients were reported to be AF free, follow-up was limited to 3 months (78). These types of techniques are frequently used in hybrid ablation (see below).

We have previously noted the report from Edgerton et al. on the efficacy of a minimally invasive surgical approach to treat AF that combined pulmonary vein antral isolation with targeted partial autonomic denervation (47). The authors reported performance of pulmonary vein antral electrical isolation (with confirmation of block) and partial autonomic denervation in 83 AF patients. The results for 57 patients (39 paroxysmal, 18 persistent/longstanding persistent) with ≥6 months of follow up and pacemaker interrogation (9), 14- to 21-day event monitors (24), or a 24-h Holter monitor (24) were reported. Success was defined as no episodes of atrial fibrillation >15 s in duration recorded via these monitoring modalities. Treatment was successful in 32 of 39 (82.1%) patients with paroxysmal atrial fibrillation and 10 of 18 (55.6%) with persistent/long-standing persistent atrial fibrillation (47). We are unaware of additional evaluation of this technique.

In a 2012 consensus document (79) the primary AF surgical indication was the presence of symptomatic AF, refractory or intolerant to at least one Class I or Class III anti-arrhythmic drug. Currently, most patients have also had ≥1 unsuccessful catheter ablation procedure prior to surgical referral, unless they have a strong preference for cure with a single procedure (48).

In 2015, Gillinov et al. reported results from 260 patients with persistent or long-standing persistent AF who underwent mitral-valve surgery and were randomly assigned to undergo concomitant surgical ablation or no ablation (control group) during their operation. Patients in the ablation group were further randomized to a biatrial maze procedure or pulmonary vein isolation. Freedom from AF was not significantly different between the two ablation techniques. More patients in the ablation group than the control group were free from AF at both 6 and 12 months (63.2 vs. 29.4%, P < 0.001). Ablation was associated with more permanent pacemaker implantations than no ablation (P = 0.01) (80).

Responses to this study suggested the low rate of success of the biatrial maze procedure indicated either incorrectly performed surgery or inadequate lesions. It was also noted that maze procedures include ablation of the coronary sinus, and that such ablation was not mentioned (81).

In terms of efficacy, it should be noted that although lesion sets were standardized, energy sources were not (80, 82). Surgeons were permitted to use a combination of bipolar and unipolar radiofrequencies and cryothermy (82). Pison et al. pointed out that bipolar pulmonary vein isolation was performed in only 53 patients, as compared with unipolar ablation or cryoablation in 110 patients and noted that unipolar and cryoablation energies are less effective in creating transmural lesions and are associated with pulmonary vein reconnection. Therefore, they raised was associated question of whether greater use of bipolar radiofrequency ablation would have resulted in higher success rates (83). Gillinov et al. (82) responded that bipolar radiofrequency was used in 43% of the 67 patients who underwent pulmonary vein isolation and greater freedom from atrial fibrillation was not observed in this small group.

The validity of finding that the maze procedure caused patients to need more postoperative pacing was also questioned (81). The authors (82) admitted surprise that the need for pacing was so high (17%). They had raised the possibility that it was caused by valve replacement, multivalve surgical procedures (~50% of patients who underwent ablation had multivalve surgery) or that >50% of the patients who underwent ablation were 70 years of age or older, all factors that increase the risk of postoperative atrioventricular block (80). They also speculated that it could be due to the biatrial maze (82). A previous trial noted that addition of right atrial lesions to an extended left atrial lesion set did not improve efficacy but did increase the rate of pacemaker placement (to 16.5%). However, in that study cohort, the increase was due to sinus node dysfunction (84).

In the ACC/AHA/HRS 2014 guidelines, surgical ablation was only recommended for: (a) selected patients with AF undergoing cardiac surgery for other indications (Recommendation Class IIa, level of evidence C) and (b) selected patients with highly symptomatic AF not well managed with other approaches (85, 86). The 2017 HRS/EHRA/ECAS/APHRS/SOLAECE expert consensus statement on catheter and surgical ablation of atrial fibrillation (48) provides a more extensive list of surgical AF ablation indications (see Table 1).

**Table 1:** Indications for surgical ablation of atrial fibrillation.

**Clinical scenario**	**AF type**	**Recommendation**	**Class**	**LOE**
(A) Indications for concomitant open (such as mitral valve) surgical ablation of atrial fibrillation				
Symptomatic AF refractory or intolerant to at least one Class I or III antiarrhythmic medication	Paroxysmal AF	Surgical ablation is recommended	I	B-NR
	Persistent AF	Surgical ablation is recommended	I	B-NR
	Long-standing persistent AF	Surgical ablation is recommended	I	B-NR
Symptomatic AF prior to initiation of antiarrhythmic therapy with a Class I or III antiarrhythmic medication	Paroxysmal AF	Surgical ablation is recommended	I	B-NR
	Persistent AF	Surgical ablation is recommended	I	B-NR
	Long-standing persistent AF	Surgical ablation is recommended	I	B-NR
(B) Indications for concomitant closed (such as CABG and AVR) surgical ablation of atrial fibrillation				
Symptomatic AF refractory or intolerant to at least one Class I or III antiarrhythmic medication	Paroxysmal AF	Surgical ablation is recommended	I	B-NR
	Persistent AF	Surgical ablation is recommended	I	B-NR
	Long-standing persistent AF	Surgical ablation is recommended	I	B-NR
Symptomatic AF prior to initiation of antiarrhythmic therapy with a Class I or III antiarrhythmic medication	Paroxysmal AF	Surgical ablation is reasonable	IIa	B-NR
	Persistent AF	Surgical ablation is reasonable	IIa	B-NR
	Long-standing persistent AF	Surgical ablation is reasonable	IIa	B-NR
(C) Indications for stand-alone and hybrid surgical ablation of atrial fibrillation				
Symptomatic AF refractory or intolerant to at least one Class I or III antiarrhythmic medication	Paroxysmal AF	Stand-alone surgical ablation can be considered for patients who have failed one or more attempts at catheter ablation and also for those who are intolerant or refractory to antiarrhythmic drug therapy and prefer a surgical approach, after review of the relative safety and efficacy of catheter ablation vs. a stand-alone surgical approach.	IIb	B-NR
	Persistent AF	Stand-alone surgical ablation is reasonable for patients who have failed one or more attempts at catheter ablation and also for those who prefer a surgical approach, after review of the relative safety and efficacy of catheter ablation vs. a stand-alone surgical approach.	IIa	B-NR
	Long-standing persistent AF	Stand-alone surgical ablation is reasonable for patients who have failed one or more attempts at catheter ablation and also for those who prefer a surgical approach, after review of the relative safety and efficacy of catheter ablation vs. a stand-alone surgical approach.	IIa	B-NR
Patients being considered for hybrid surgical AF ablation		It might be reasonable to apply the indications for stand-alone surgical ablation described above to patients being considered for hybrid surgical AF ablation.	IIb	C-EO

AF, atrial fibrillation; LOE, Level of evidence; B-NR, Data derived from non-randomized trials; EO, Expert Opinion.

Adapted from Calkins et al. (48) with permission.

## Direct comparisons of surgical and catheter ablation of AF

The 2012 FAST trial included 124 patients with antiarrhythmic drug–refractory AF with left atrial dilatation and hypertension (42 patients, 33%) or prior failed catheter ablation (82 patients, 67%) were randomized to catheter ablation (63 patients) or video-assisted thoracoscopic surgical ablation. Catheter ablation included antral pulmonary vein isolation ± additional lines. Surgical ablation included bipolar radiofrequency isolation of the bilateral pulmonary veins, ganglionated plexi ablation, and left atrial appendage excision ± additional lines.

Six- and 12-month follow-up was performed by ECG and 7-day Holter recording. The primary end point, freedom (without antiarrhythmic drugs) from >30 s of left atrial arrhythmia after 12 months, was 36.5% in the catheter ablation group and 65.6% in the surgical ablation group (P = 0.0022). The primary safety end point, significant adverse events during the 12-month follow-up, were higher for surgical ablation than catheter ablation (34.4 vs. 15.9%; P = 0.027), primarily due to procedural complications such as pneumothorax, major bleeding, and the need for a pacemaker. In the catheter ablation group, one patient died at one month from a subarachnoid hemorrhage (87).

Phan et al. performed a systematic review and meta-analysis of eight studies comparing catheter ablation to video-assisted thoracoscopic surgical ablation. Twelve-month, freedom from AF off antiarrhythmic drugs (AAD) was significantly higher in the surgical cohort versus the catheter ablation cohort (78.4 vs. 53%; P < 0.0001). Twelve-month freedom from AF on antiarrhythmic drugs results for surgical ablation were also superior (82.6 vs. 45.7%; P < 0.00001). Freedom from AF/arrhythmias at 12-months off-AAD was compared for paroxysmal AF and persistent AF. Surgical ablation results were superior for paroxysmal AF (82.0 vs. 62.5%; P = 0.04). Surgical ablation results were also better for persistent AF (74.4 vs. 51.1%; P = 0.002). Not surprisingly, complications were significantly higher in the surgical ablation group (28.2 vs. 7.8%; P = 0.0003) (88).

The SCALAF trial directly enrolled 52 patients who had failure of ≥1 class I or III antiarrhythmic drugs. The investigators compared minimally invasive thoracoscopic PVI with left atrial appendage ligation (surgical MIPI) versus percutaneous catheter ablation comprised of PVI as primary AF treatment. The follow-up strategy in this study was continuous rhythm monitoring with an ILR in all patients. Single-procedure arrhythmia-free survival without antiarrhythmic drugs after 2 years of follow-up was higher in the catheter ablation group compared to surgical ablation (2-year Kaplan-Meier event rate estimates, 56.0 and 29.2%, respectively), however this difference did not reach statistical significance. Procedure-related adverse events during follow-up occurred more often in the surgical group (P = 0.046). This was primarily due to major complications (P = 0.029). The authors concluded catheter ablation was non-inferior to surgical ablation for treatment of paroxysmal and early persistent AF (89). Patients who underwent catheter ablation reported significantly fewer physical problems and less body pain 3 months post treatment (90).

Long-standing persistent AF has been associated with suboptimal catheter ablation outcomes. The CASA-AF trial randomized 120 patients with longstanding persistent AF to surgical or catheter ablation. The primary outcome was single procedure freedom from AF/atrial tachycardia (≥30 s) at 12 months without antiarrhythmic drugs. Secondary outcomes included clinical success (≥75% reduction in AF/atrial tachycardia burden); procedure-related serious adverse events; symptom changes and quality-of-life scores; as well as cost-effectiveness.

Catheter ablation was performed with point-by-point radiofrequency ablation including PVI, roof and inferior lines to create a posterior wall box lesion as well as lateral mitral isthmus and cavotricuspid isthmus lines. Video-assisted thoracoscopic AF surgery included pulmonary vein isolation, ganglionic plexus ablation, followed by linear roof and inferior lines to create a posterior wall box lesion. The LAA was excluded using the AtriClip^®^ LAA excluder system (AtriCure Mason, Ohio, USA). At 12 months, freedom from AF/AT was not significantly different in the two groups (26% in the surgical cohort vs. 28% in the catheter ablation cohort). Reduction in AF/AT burden ≥75% was also not significantly different in the two groups (67% in the surgical cohort vs. 77% in the catheter ablation cohort). Somewhat surprisingly, procedure-related serious adverse events occurring within 30 days of intervention were not significantly different in the two groups.

However, surgical ablation was more expensive and provided fewer quality-adjusted life-years compared to catheter ablation (*P* = 0.02) (91). Interestingly, despite similar low success rates, catheter ablation provided greater symptom improvements and accrued significantly more quality-adjusted life-years during follow-up (91).

Currently, surgical ablation has a Class I recommendation for AF patients undergoing concomitant open surgery, and a Class IIa for patients with AF undergoing closed surgery (coronary artery bypass grafting, aortic valve replacement). Catheter ablation currently has a Class IA recommendation for symptomatic paroxysmal AF refractory to antiarrhythmic medication. For persistent and longstanding persistent AF (>1 year), the recommendations are IIa, and IIb respectively. For persistent or long-standing persistent AF, posterior wall isolation and ablation of non PV triggers also has a Class IIb recommendation (92).

We believe that the take home message from these trials is that early intervention is crucial to success.

## Hybrid AF ablation

In 2011, Krul et al. described a “hybrid” approach to AF ablation similar to what was described by Edgerton et al. (47) for thoracoscopic AF ablation. Briefly, the procedure consisted of isolating the pulmonary venous antra, left atrial ablation lines, and ganglionated plexus (GP) ablation. The ganglionic plexi were located via high frequency pacing and ablated with bipolar radiofrequency energy. A rubber banding was placed under the pulmonary venous antrum. An AtriCure Isolator Transpolar Clamp (AtriCure Mason, Ohio, USA) was then connected to the rubber banding and gently positioned around the antrum. Isolation of the pulmonary venous antra was accomplished via application of bipolar radiofrequency energy to the clamps around each PV antrum. A standard decapolar electrophysiology catheter was positioned behind the left atrium to record electrograms pre- and post-procedure to confirm isolation. In patients with persistent AF or longstanding persistent AF additional ablation lines connected the pulmonary veins and isolated the posterior LA wall. Entrance and exit block of the “box” was confirmed with pacing maneuvers. The entire procedure was epicardial (93).

In 2011, during the same month, Mahapatra et al. reported results from 45 patients who underwent sequential surgical epicardial-catheter endocardial ablation for persistent and long-standing persistent AF vs. catheter ablation alone. All patients had a failed previous catheter ablation. The sequential catheter ablation was performed 4.3 ± 1.3 days after the surgical procedure. After a mean follow-up of 20.7 ± 4.5 months, 86.7% (13/15) of sequential patients were free of atrial arrhythmias and off antiarrhythmic drugs, compared to 53.3% (16/30) of catheter-alone patients (P = 0.04) (94).

In 2012, Pison et al. reported a cohort of 26 consecutive AF patients who underwent simultaneous hybrid thoracoscopic surgical and transvenous catheter ablation followed for up to 1 year. The epicardial lesions were not transmural in 23% of the patients, and endocardial touch-up was needed. The procedure was deemed feasible and safe. The single-procedure success rate was 83% at 1 year (95). Thus, hybrid ablation in its current form was shown to be effective.

A 2015 systematic review compared the efficacy and safety of the Cox-Maze procedure (with cardiopulmonary bypass support [CPB]), beating-heart epicardial ablation, and the hybrid procedure. At 1-year, sinus rhythm restoration rates were 93, 80 and 70%, and sinus restoration without anti-arrhythmic medication was 87, 72 and 71%, for Cox-Maze, epicardial and hybrid procedures, respectively. The minimally invasive Cox-Maze procedure with CPB had important safety advantages in conversion to sternotomy and the lowest incidence of reoperation for major bleeding (96).

Despite the apparent superb results of surgical AF ablation, its morbidity (especially for stand-alone procedures) has limited widespread use (97). Minimally invasive thoracoscopic surgery may exceed AF catheter ablation results, but limitations in creation of transmural roof and floor lesions on the left atrial posterior wall can reduce its efficacy compared to open CPB surgery. Despite use of various ablation strategies for persistent AF, single procedure success rates have ranged from ~20–60% (98, 99). For the combination of persistent and longstanding persistent AF, efficacy rates are ~30–40%. Efficacy seems somewhat dependent on the procedural approach used. Long-standing persistent AF may be effectively treated with a composite of extensive index catheter ablation, repeat procedures, and/or pharmaceuticals (98–100). Rostock et al. reported that after 2.3 ± 0.6 ablation procedures in 395 patients, 312 (79%) were free of arrhythmia (with concomitant antiarrhythmic treatment in 38%) at mean follow-up of 15 ± 9 months after their last procedure (100). A 2017 report (not confined to persistent and longstanding persistent AF) found that patients with an additional ablation had $39,409 more in costs the following year (101).

These results, as well as the knowledge that thoracoscopic surgical and catheter ablation of AF may result in incomplete isolation of the pulmonary veins and the posterior left atrial wall, spurred further development of hybrid strategies combining these approaches (46, 98–100).

Hybrid AF ablation employs subsets of the Cox-Maze IV minimally invasive epicardial lesion sets followed by endocardial catheter ablation to fill in non-transmural gaps between ablation lesions and address additional atrial reentrant circuits. Unfortunately, similar to surgical approaches, (68, 77, 82, 97) lack of uniformity remains in the procedural approach including (but not limited to) pericardioscopic vs. thoracoscopic approaches, energy sources, the lesion set applied, timing of the surgical and catheter components, management of the left atrial appendage (LAA), and the medical management of these patients (97).

There have been superior outcomes using a bilateral thoracoscopic ablation compared with the pericardioscopic approach including lower rates of morbidity and mortality. The bilateral thoracoscopic approach excludes (e.g., clipping, ligation, or excision) the LAA in patients with persistent or long-standing persistent AF. LAA exclusion has potential to reduce their lifetime risk of stroke as well as electrically isolate any AF triggers from the LAA (97, 102). Benefit from surgical LAA ligation/excision is discussed in more detail under stroke/thromboembolic prevention.

As noted above, hybrid ablation studies have plagued by lack of uniformity in approaches, lesion sets, catheter ablation timing relative to the surgical approach, and follow-up protocols (92).

CONVERGE (Convergence of Epicardial and Endocardial Ablation for the Treatment of Symptomatic Persistent AF), a multicenter randomized controlled trial, evaluated the safety of hybrid ablation and compared its efficacy to catheter ablation for treatment of persistent and long-standing persistent AF (103). Epicardial and endocardial procedures were performed in a single setting (103).

In this industry sponsored trial (AtriCure, Mason, OH, USA), patients (ages 18–80 years) with symptomatic persistent AF intolerant of or refractory to ≥1 class I/III antiarrhythmic agent and a left atrial diameter ≤ 6 cm were randomized in a 2:1 ratio to Hybrid Convergent or endocardial catheter ablation. Long-standing persistent AF was present in 42% of patients enrolled (103).

In contrast to “preferred” methods noted above, epicardial ablation was performed via pericardioscopic access with the vacuum-assisted, unipolar radiofrequency device (EPi-Sense, AtriCure, Mason, OH, USA). Endocardial ablation was subsequently performed using an irrigated radiofrequency catheter aiming to assure complete PVI, close gaps in linear lesions, and create cavotricuspid isthmus block (103). If AF termination did not occur, CFAEs could be targeted at the operator's discretion (103).

Following a 3-month blanking period, the primary end point was freedom from AF/atrial flutter (AFL)/atrial tachycardia (AT) in the absence of class I/III antiarrhythmic agents, except previously failed or intolerable antiarrhythmic agents as long as the dose was not increased. Secondary endpoints included a 90% reduction in AF burden as well as freedom from AF (only) in the absence of dose increases or new class I/III antiarrhythmic agents. The targeted follow-up duration was 1 year.

Hybrid Convergent ablation was superior to endocardial catheter ablation in persistent and long-standing persistent AF. Freedom from atrial arrhythmia in the absence of new/increased dosage of previously failed class I/III antiarrhythmic agents was 67.7 vs. 50.0% (P = 0.036). Success rates off antiarrhythmic drugs were 53.5% vs. 32.0% (P = 0.0128). Additional follow-up at 18 months (via 7-day Holter monitor) revealed that 74% of Hybrid Convergent patients achieved ≥90% AF burden reduction compared to 55% who only underwent endocardial catheter ablation. Major adverse events were more common in the Hybrid Convergent group (8/102, 7.8 vs. 0/51, 0%, P = 0.0525) although this difference did not quite reach statistical significance (104).

The “Pivotal Study Of A Dual Epicardial and Endocardial Procedure (DEEP) Approach for Treatment of Subjects With Persistent or Long Standing Persistent Atrial Fibrillation With Radiofrequency Ablation” aims to establish the safety and efficacy of a combined epicardial and endocardial ablation procedure for patients with persistent AF or longstanding persistent AF using the AtriCure Bipolar System and AtriClip^®^ PRO LAA Exclusion System (AtriCure, Mason, OH, USA) in an endoscopic or open ablation procedure, followed by an endocardial mapping and ablation procedure performed with available RF based, irrigated, power controlled, catheters and endocardial only lesions. The endocardial procedure will be added 90 days after the epicardial surgical procedure. The initial results are expected to be reported in late 2022 (105).

In addition to DEEP, 7 other hybrid ablation studies are underway in Europe and Asia. It is hoped that these ongoing clinical trials will lead to standardization of hybrid protocols with respect to lesions sets, catheter ablation timing in relationship to surgery as well as follow up protocols (92).

## AF and pharmacologic stroke/thromboembolic prevention

AF is associated with a 4-to-5-fold increase in the risk of stroke. An estimated 15% of all strokes are caused by AF and this proportion increases substantially with age (104, 105).

AF-related stroke is associated with greater early severity, disability, fatality, and cost compared with non-AF stroke (105–110). Stroke patients with AF are at high risk of death in the first 28–30 days post event as well as during subsequent years after their first acute stroke (108, 109, 111). In a prospective, population-based study, only 14% of patients with AF-related strokes were alive and independent at 5 years and 26% required nursing-home care (107). However, in this study, only 32% of ischemic AF-stroke survivors were prescribed oral anticoagulant (OAC) therapy with warfarin after treatment of their index stroke, and only 48% were on OAC treatment at 5 years (107).

Among patients with AF treated with OAC, annual stroke risk is lowered by approximately two-thirds. Although the risk of systemic embolism or stroke generally outweigh the bleeding risk in AF patients, analysis of individual risk and benefit of anticoagulation is requisite. The CHA_2_DS_2_-VASc score (112, 113) (Table 2A) generally outperforms other risk assessment scales, particularly in identifying low risk individuals (CHA_2_DS_2_-VASc score of 0 in males or 1 in females) in whom anticoagulation is generally not recommended.

**Table 2:** CHA_2_DS_2_-VASc and HAS-BLED risk scores.

**A**		**B**	
**CHA**_2_ **DS**_2_ **-VASc stroke risk score**		**HAS-BLED risk score**	
**Characteristic**	**Score**	**Characteristic**	**Score**
Congestive heart failure/LV dysfunction	1	Hypertension (systolic BP >160)	1
Hypertension	1	Abnormal renal^*^ (1 point) or liver^**^ (1 point) function	1 or 2
Aged ≥ 75 years	2	Stroke	1
Diabetes mellitus	1	Bleeding	1
Stroke/TIA/TE	2	Labile INRs	1
Vascular disease (prior MI, PAD, or aortic plaque)	1	Elderly (age > 65)	1
Aged 65–74 years	1	Drugs: antiplatelet agents or NSAIDS (1 point) Alcohol ≥ 8 drinks per week (1 point)	1 or 2
Sex category (female gender)	1		
Maximum score	9	Maximum score	9

* Chronic dialysis or renal transplantation or serum creatinine ≥2.26 mg/dl.

** Chronic hepatic disease, bilirubin >2x upper limit of normal, in association with aspartate transaminase/alanine transaminase/alkaline phosphatase > 3x upper limit of normal.

Adapted from reference (113) with permission.

Chronic oral anticoagulation is recommended when the CHA_2_DS_2_-VASc score is ≥2 in males or ≥3 in females. When the CHA_2_DS_2_-VASc score is 1 in males or 2 in females, clinical judgment and bleeding risk assessment are even more pivotal. Age 65–74 years is the strongest risk factor among the risks conferring one CHA_2_DS_2_-VASc score point. Chronic oral anticoagulation is recommended in this specific cohort. In patients under age 65 with CHA_2_DS_2_-VASc scores in this range due to other risk factors who have a very low AF burden, it is reasonable to forgo anticoagulation unless the patient strongly prefers drug therapy.

The HAS-BLED risk score (Table 2B) generally outperforms other bleeding risk assessment scales (112, 113). The most important predictors of major bleeding (including intracranial hemorrhage) are older patient age, excessive warfarin anticoagulation (INR> 3.0), and prior stroke. Bleeding risk is significantly higher in the presence of thrombocytopenia or known coagulation defect(s), prior severe bleeding on an oral anticoagulant, active bleeding or recent surgery, combination use of anticoagulant and antiplatelet therapy, aortic dissection and malignant hypertension (112). Patients with elevated CHA_2_DS_2_-VASc scores and significant bleeding risk(s) should be considered as candidates for percutaneous left atrial appendage occlusion (see below).

It should be noted that seeking AF catheter ablation to avoid long-term oral anticoagulation is not recommended. Current recommendations suggest that anticoagulation should be continued at least 2 months post ablation and thereafter guided by CHA_2_DS_2_-VASc scores (48).

Phase I of the international Global Registry on Long-Term Oral Anti-thrombotic TReatment In PAtients With Atrial Fibrillation (Gloria-AF) aimed to characterize the newly diagnosed non-valvular AF patients at risk for stroke and the selection of antithrombotic treatment for stroke prevention in a real-world setting prior to approval of the first direct oral anticoagulant (dabigatran) for prevention of strokes and systemic emboli in non-valvular AF patients (114, 115).

In the RE-LY trial (116), dabigatran 150 mg twice daily was superior to warfarin for preventing stroke/systemic embolism and ischemic stroke in non-valvular AF patients. Major bleeding rates were similar to warfarin. Dabigatran 110 mg twice-daily was non-inferior to warfarin for prevention of stroke/systemic embolism, with substantially less major bleeding. Both dabigatran doses resulted in less intracranial hemorrhage compared with warfarin (116). Gloria-AF Phase II confirmed the sustained efficacy and safety of dabigatran over 2 years of observation in clinical practice (117).

There are now four FDA approved and available direct oral anticoagulants (DOACs; AKA non-vitamin K oral anticoagulants [NOACs]) for use in patients with non-valvular AF. A fifth DOAC, betrixaban (approved for venous thrombosis prophylaxis) has been removed from the market for business reasons (118). Dabigatran is a direct thrombin inhibitor. Rivaroxaban, edoxaban, and apixaban are factor Xa inhibitors. There have been four randomized controlled trials comparing DOACs with warfarin (119). These trials (116, 120–122) consistently provided evidence of at least non-inferiority for the combined endpoint of stroke or systemic embolism.

Ruff and colleagues performed a meta-analysis of 71,683 participants included in the RE-LY, ROCKET AF, ARISTOTLE, and ENGAGE AF-TIMI 48 trials. The main outcomes analyzed were stroke and systemic embolic events, ischemic stroke, hemorrhagic stroke, myocardial infarction, major bleeding, gastrointestinal bleeding, intracranial hemorrhage, and all cause mortality. In these trials, 42,411 participants received a direct oral anticoagulant and 29,272 received warfarin. Compared with warfarin, DOACs significantly reduced stroke or systemic embolism by 19% (P < 0.0001), predominantly due to a reduction in hemorrhagic stroke (P < 0.0001). DOACs also reduced intracranial hemorrhage (P < 0.0001) and mortality (P < 0.0003). Use of DOACs increased gastrointestinal bleeding (P = 0.04). Overall major bleeding was similar between the two treatment modalities (123). DOACs are recommended as first-line therapy for prevention of ischemic stroke in AF patients by both European and North American guidelines (119, 124).

A 2022 meta-analysis collected from the same population demonstrated that use of standard-dose DOACs results in decreased incidence of stroke, death, and intracranial hemorrhage with no difference in major bleeding. The authors suggested that younger patients and those with lower body weight may derive greater benefit in regard to major bleeding from standard-dose DOACs vs. warfarin (125).

Eligibility for DOACs requires meeting the criteria for non-valvular AF. The distinction between non-valvular and valvular AF can be confusing. Non-valvular AF does not mean complete absence of valvular heart disease. For anticoagulant management purposes non-valvular AF is present when moderate-to-severe mitral stenosis or a mechanical heart valve are absent.

Valvular AF refers to its presence in the setting of moderate-to-severe mitral stenosis (possibly requiring surgery) or when a mechanical heart valve is present and is an indication for long-term warfarin anticoagulation (119). For valvular AF patients treated with warfarin an international normalized ratio (INR) of 2.5–3.5 is recommended.

Although DOAC therapy does not require regular INR monitoring it is pivotal to understand that reduced clearance increases bleeding risk and enhanced metabolism increases risk of stroke or systemic embolization. An analysis of 14,865 AF patients with renal dysfunction demonstrated that DOAC overdosing was associated with a 2.2-fold increased risk of major bleeding (126, 127). Apixaban underdosing was associated with 4.9-fold increased risk of stroke (126). There was no significant increase in stroke with underdosing of rivaroxaban or dabigatran (126).

All DOACs have some level of renal clearance (80% for dabigatran, 50% for edoxaban, 35% for rivaroxaban, and 27% for apixaban). Dose reduction of DOACs is indicated in patients with non-valvular AF and significant renal impairment. Clinicians should carefully review renal function prior to initiating DOAC treatment (126).

Dabigatran-treated patients with an eGFR < 30 ml/min/1.73 m^2^ would qualify for dose reduction to 110 mg in Europe, but this dose was not approved by the U.S. Food and Drug Administration (FDA) and 75 mg twice daily is recommended for creatinine clearances (CrCL) between 15 and 30 ml/min. The indication for apixaban dose reduction requires two of the following three criteria: age ≥ 80 years, weight ≤ 60 kg, and serum creatinine level ≥ 1.5 mg/dl. Apixaban's renal indication for dose reduction correlates closely with eGFR < 50 ml/min/1.73 m^2^, which is the renal indication for rivaroxaban dose reduction to 15 mg daily. The recommended dose of edoxaban is 60 mg daily. A reduced dose of 30 mg daily is recommended for patients with CrCL 15–50 ml/min. Edoxaban blood levels are lower in patients with better renal function (~50% of an edoxaban dose is excreted by the kidneys). There is an inverse relationship between edoxaban blood levels and CrCL (128). Trough levels are reduced, and an increased risk of ischemic stroke compared to warfarin has been demonstrated (123, 126, 128, 129). In the United States, the FDA issued a Boxed Warning that edoxaban (brand name: SAVAYSA) should not be used in patients with a CrCL > 95 ml/min. In contrast, the European Medicines Agency (EMA) and the Korean Ministry of Food and Drug Safety (MFDS) advise that edoxaban should only be used in patients with NVAF and high CrCL after careful evaluation of individual thromboembolic and bleeding risk. In Japan, the Pharmaceuticals and Medical Devices Agency (PMDA) has not issued either a warning or cautions (130).

In addition to knowing adjusted renal doses, prescribers need to be aware of drug-drug interactions (DDIs) with DOACs. The most common DOAC DDIs involve drugs metabolized by the cytochrome P450 enzymes (primarily CYP3A4) and/or the transporter permeability glycoprotein (P-gp). Isolated P-gp inducers are believed to have little clinical impact on DOAC dosing, however strong data is lacking (131). P-gp inducers frequently also induce CYP3A4. Other transport mechanisms have also been implicated. Indications for DOAC dose adjustments and contraindications are summarized in Table 3.

**Table 3:** DOACs: drug interactions.

**DOAC**	**Mechanism of action**	**Dosing**	**↓Effect**	**↑Effect**	**Drug-drug interactions**	**Management recommendations**
Dabigatran	Direct thrombin inhibition	150 mg p.o. b.i.d. CrCl > 30 ml/min 110 mg p.o. b.i.d. if: Age ≥ 80 Concomitant use of verapamil Increased bleeding risk 75 mg p.o. b.i.d. (see Management recommendations)	P-gp inducers can decrease dabigatran effect	P-gp inhibitors can increase dabigatran effect	Dronedarone	Administer 2 h before dronedarone Reduce dose to 75 mg twice daily for CrCl 30–50 ml/min Avoid use if CrCl < 30 ml/min
					Amiodarone	Safe if CrCl > 50 ml/min Avoid combination if CrCl < 30 ml/min
					Verapamil	Avoid use if CrCl < 30 ml/min
Rivaroxaban	Factor Xa inhibition	20 mg p.o. qd 15 mg p.o. qd (CrCl < 50 ml/min) CrCl < 15 ml/min. or dialysis: not recommended	Strong CYP3A4 inducers and/or P-gp inducers can decrease rivaroxaban effect	Strong dual CYP3A4 and P-gp inhibitors can increase rivaroxaban effect	Dronedarone Amiodarone Verapamil Diltiazem	Avoid combination if CrCl < 80 ml/min
Apixaban	Factor Xa inhibition	5 mg p.o. b.i.d. 2.5 mg p.o. b.i.d. (if at least 2 of 3: age ≥ 80, creatinine ≥ 1.5 mg/dl, weight < 60 kg)	Strong CYP3A4 inducers and/or P-gp inducers can decrease apixaban effect	Strong dual CYP3A4 and P-gp inhibitors can increase apixaban effect Strong single CYP3A4 inhibitors can also increase apixaban effect	Enzyme inducers such as phenytoin, carbamazepine, primidone, phenobarbital, rifampin, St. John's wart	Avoid use; consider warfarin
Edoxaban	Factor Xa inhibition	CrCl > 95 ml/min: not recommended (U.S. only) CrCl 51–95 ml/min: 60 mg p.o. qd CrCl 15–50 ml/min: 30 mg p.o. qd	P-gp inducers can decrease edoxaban effect	P-gp inhibitors can increase edoxaban effect	Dronedarone	Reduce dose by 50% to 30 mg p.o. qd

Adapted from Hindricks et al. (124), Wiggins et al. (127), and Manning et al. (131) with permission.

A recent meta-analysis included 21 studies from 2010 to 2018, with a total of 9,758,637 patients and 197,483 had AF. Eleven studies were European, 3 were North American, 4 Asian, 1 Oceanian (i.e., Australia, Fiji, Kiribati, the Marshall Islands, Micronesia, Nauru, New Zealand, Palau, Papua New Guinea, Samoa, the Solomon Islands, Tonga, Tuvalu, and Vanuatu), 1 South American and 1 study with both worldwide and North American data (132). The prevalence of oral anticoagulation use among eligible AF patients rose from 42% in 2010 to 78% 2018. Global initiation of oral anticoagulation among newly eligible AF patients rose from 43 to 75% during this time frame. The proportion of DOACs among this group rose from 0 to 68% while the proportion starting vitamin K antagonists fell from 42 to 6%. Although trends tended to be similar in different regions DOAC uptake occurred earlier in North America. Prevalence of oral anticoagulant use was lower in Asia (data from China was lacking) compared to North America and Europe. This is similar to what was noted in the Garfield-AF registry where, from 2010 to 2013, Asian use of vitamin K antagonists in AF patients lagged behind other regions of the world (37.8 vs. 53.3%) and time spent in the therapeutic range of 2.0–3.0 was considerably lower (31.1 vs. 54.1%) (132, 133).

Although most AF patients with an anticoagulation indication should be prescribed a DOAC, continued use of warfarin remains reasonable for current recipients with an annual time in the therapeutic (2.0–3.0) range ≥70%. Warfarin candidates also include patients unlikely to comply with twice daily dosing of dabigatran or apixaban and are unable to use rivaroxaban or edoxaban due to intolerance or contraindications. In addition, lack of insurance coverage for DOACs (in the United States) may make their costs prohibitive and result in warfarin becoming patients' sole viable option (131).

Warfarin's anticoagulation effects occur via reduced synthesis of vitamin K-dependent clotting factors (II, VII, IX, and X) and anticoagulant proteins C and S. Multiple herbal products and medications can potentiate or inhibit warfarin effects. Warfarin's hepatic metabolism and protein binding are the most common mechanisms for the occurrence of drug-drug interactions. Warfarin is metabolized by the cytochrome P450 system via CYP 2C9, 1A2, and 3A4. Major interactions may occur with concomitant use of the CYP inhibitors metronidazole, trimethoprim-sulfamethoxazole, and ciprofloxacin resulting in increased INRs. Rifampin is a CYP inducer and higher warfarin doses may be required to maintain therapeutic levels during concomitant use. Short term use of phenytoin may increase the INR via displacement of warfarin from protein binding sites.

Long term phenytoin use may decrease the INR since it is a CYP inducer (134, 135). Clinicians should consider consulting a drug information source when starting new medications in warfarin recipients and should always do so when suspecting a drug-drug interaction with warfarin (135).

The interaction between warfarin and amiodarone is particularly important because they are commonly used together in the management of AF patients (136). Amiodarone inhibits plasma clearance of warfarin, thereby increasing its anticoagulant action. This effect appears to be mediated by competitive inhibition of hepatic cytochrome P450, family 2, subfamily C, polypeptide 9 gene (CYP2C9) and the vitamin K epoxide reductase subunit 1 gene (VKORC1). However, hyperthyroidism afflicts ~0.9–10% of amiodarone recipients. It has been our experience that the most common clinical presentation is the sudden recrudescence of previously controlled atrial and/or ventricular tachyarrhythmias. In patients with recurrent AF, prevention of thromboembolic events may be particularly problematic. Although thyrotoxicosis modifies the balance between coagulation and fibrinolysis and exerts a procoagulant effect increasing the risk of thromboembolism; in patients receiving warfarin, thyrotoxicosis has been associated with increased warfarin sensitivity (regardless of the thyroid disorder's etiology). Hyperthyroid patients exhibit an exaggerated depression in functional clotting factors (II, VII. IX and X) in response to warfarin and accentuation of prothrombin times. Amiodarone's long half-life prevents any immediate benefit from drug discontinuation. Thyrotoxicosis may take as long as 8 months to subside after amiodarone is discontinued (135, 137).

Green leafy vegetables are high in vitamin K and may inhibit warfarin's anticoagulant efficacy. Nevertheless, they can be consumed in moderation. While cranberry juice has been suggested to potentiate warfarin, the evidence is questionable and moderate consumption seems to be fine (138). Alcohol acutely inhibits warfarin metabolism, however, chronic use may induce hepatic metabolism and decrease INR (135).

## Special circumstances

Although cardioversion of AF to maintain sinus rhythm has previously been described as “A Road to Nowhere” (139), biphasic electrical (direct current) cardioversion terminates AF in over 90% of cases. It is the treatment of choice in severely hemodynamically compromised individuals with new-onset AF (140). This procedure remains widely used in AF patients when a rhythm control strategy is pursued (140).

Peri-procedural thromboembolic event rates range between 1.1–2% in patients insufficiently or not anticoagulated and 0.00–0.8% in patients sufficiently anticoagulated (140, 141). Although (traditionally) patients with AF lasting < 48 h were believed to have a low risk of thromboembolic events post-cardioversion (~0.7%), the risk of thromboembolic events increases with higher CHA_2_DS_2_-VASc scores (140). In 2016, two large studies clarified this issue. In a study from the Cleveland Clinic, 567 cardioversions in 484 patients were performed without therapeutic anticoagulation and 898 cardioversions in 709 patients were performed on therapeutic anticoagulation. There were six neurologic events (1.06%) in the group without therapeutic anticoagulation and 2 (0.22%) in the group on therapeutic anticoagulation (P = 0.03) No events occurred in patients with CHA_2_DS_2_-VASc scores < 2 (142). In the FinCV Study, a retrospective, multicenter study of 3,143 patients, who underwent 7,660 cardioversions for acute (within 48 h) AF, thromboembolic complications increased significantly from 0.4% in patients with a CHA_2_DS_2_-VASc score of 0 to 1 to 2.3% in patients with a score of ≥5 (P < 0.001). Thromboembolic complications were significantly lower in cardioversions performed on anticoagulation (P = 0.001). Anticoagulation's preventive effect was significant in patients whose score was ≥2 (P = 0.001) (143). Multivariate analysis revealed that age per year (P < 0.001), duration of AF episode > 12 hours (P < 0.001), heart failure (P = 0.007) and female gender (P = 0.038) were significant independent predictors of thromboembolic complications in patients who were not anticoagulated. Although the vast major of thromboembolic events occur within 10 days post-procedure, we believe therapeutic oral anticoagulation should be maintained for at least 4 weeks. Current North American guidelines (119) recommend therapeutic anticoagulation for 3 weeks prior to cardioversion in patients who have been in AF for ≥48 h and that therapeutic anticoagulation should be continued for at least 4 weeks. The 2020 European guidelines recommend adherence to DOACs before and after cardioversion (Class I). These guidelines also recommend (Class IIa) that, for patients with AF > 24 h in duration, therapeutic anticoagulation should be continued for at least 4 weeks after undergoing cardioversion (124). In patients with AF of a duration ≤ 24 h and a very low stroke risk (CHA_2_DS_2_-VASc of 0 in men or 1 in women), omission of anticoagulation for 4 weeks post-cardioversion may be considered (124). DOACs and warfarin appear to be similarly effective pre and post procedure (119, 124, 140).

If a strategy of electrical cardioversion is planned without therapeutic anticoagulation for 3 weeks prior to cardioversion, transesophageal echocardiography-guided cardioversion is recommended. If sufficient anticoagulation is achieved prior to transesophageal echocardiography and no left atrial thrombus is identified, cardioversion can be performed safely. However, a left atrial thrombus is observed (most commonly in the left atrial appendage) in approximately 10% of non-valvular AF. When a thrombus is identified, therapeutic anticoagulation is recommended for ≥3 weeks before repeating transesophageal echocardiography to confirm thrombus resolution (140).

“Bridging” refers to use of unfractionated or low molecular weight heparin in patients undergoing procedures that require interruption of warfarin therapy (144). The BRIDGE Investigators conducted a randomized, double-blind, placebo-controlled trial. Following perioperative warfarin interruption (5 days prior to their procedure), patients were randomly assigned to receive bridging anticoagulation with low-molecular-weight heparin (dalteparin 100 IU/kilogram body weight) or matching placebo administered subcutaneously twice a day, from 3 days pre-procedure until 24 h prior to an elective operation or an elective invasive procedure and for 5–10 days post-procedure. Primary outcomes were arterial thromboembolism and major bleeding. Nearly 1,900 patients were enrolled. Waiving bridging anticoagulation was non-inferior to perioperative bridging with low-molecular-weight heparin for arterial thromboembolism prevention and reduced the risk of major bleeding (P = 0.005) (145).

Patient-specific high bleeding risk factors include recent (< 3 months) major bleeding, thrombocytopenia or other known bleeding diathesis, concurrent anti-platelet medication use (especially P2Y12 inhibitors; see below), an INR >1.7, a HAS-BLED score ≥3 and prior peri-procedural bleeding. Procedures associated with a high bleeding risk include urologic procedures/surgery, permanent pacemaker and ICD placement, surgery in highly vascularized organs (liver, kidney, spleen), bowel resection, cardiac, intracranial, or spinal surgery, and other major surgeries resulting in extensive tissue injury such as joint arthroplasty, cancer surgery and reconstructive plastic surgery (146).

DOACs have a much shorter time-to-onset and half-life, virtually eliminating the need for overlapping or bridging anticoagulation (146). There is a relative paucity of data to guide which procedures can be performed safely, without temporary interruption, in patients taking DOACs. However, if temporary DOAC interruption is chosen the recommended discontinuation time periods are 2–3 half-lives for low procedural bleeding risk and 4–5 half-lives for uncertain, intermediate, or high bleeding risk based on the patient's estimated creatinine clearance (147). All decisions for or against bridging therapy should balance stroke risk with the risk of bleeding (148) (see below).

Bridging therapy may be considered for AF patients with a mechanical heart valve (MHV) undergoing procedures requiring warfarin interruption. Thromboembolic risk is related to valve type and location. The highest thromboembolic risk is associated with multiple mechanical heart valves, followed by mitral mechanical valves, and the lowest is seen with aortic mechanical valves. Additional thromboembolic risks include AF, left ventricular ejection fraction < 35%, older age, hypercoagulable conditions, and a history of previous thromboembolism. The thromboembolic risk (0.08–0.36% without bridging) must be weighed against the major bleeding risk which bridging increases by an estimated 4–8% (149).

Bridging is reasonable when the INR is subtherapeutic in patients who are undergoing invasive or surgical procedures with a mechanical aortic valve and an additional thromboembolic risk factor, an older-generation mechanical aortic valve, or a mechanical mitral valve. Temporary warfarin interruption, without bridging while the INR is subtherapeutic, is recommended in patients with a bileaflet mechanical AVR and no other thrombosis risk factors who to undergo surgical or invasive procedures. Bridging is initiated when the INR falls below the therapeutic range (typically 36–48 h prior to surgery) and stopped (4–6 h for intravenous heparin and 12 h for low molecular weight heparin) pre-procedure. Warfarin is usually restarted 12–24 h post-procedure (150).

Tan and associates have suggested a pre/post-operative approach based on thromboembolic and bleeding risks. They have suggested incorporating the BleedMAP score (one point each for: history of previous bleeding, mitral MHV, active cancer, and thrombocytopenia < 150,000 cells/ml). Low bleeding risk patients would be undergoing a minor procedure or have a BleedMAP score ≤ 1. Moderate to high-risk bleeding patients would be undergoing a major procedure or have a BleedMAP score ≥2. Patients with a low thromboembolic risk would have a mechanical bileaflet aortic valve and normal sinus rhythm. Patients with a moderate to high thromboembolic risk would have a mechanical mitral valve or a mechanical aortic valve with at least one additional risk factor. Pre-operative bridging with low molecular weight heparin and post-operative bridging with either low molecular weight or intravenous heparin was recommended for individuals who fit both moderate to high-risk categories (149). To the best of our knowledge the validity of this recommendation has not been confirmed. AF ablation recipients have an increased thromboembolic risk during, immediately after, and for days to months following the procedure (48).

Aspirin has a limited role in stroke prevention for the majority of AF patients, being an inferior strategy and is not necessarily safer than the anticoagulant warfarin (151). Antiplatelet monotherapy is less effective in stroke prevention than warfarin and has a similar bleeding risk in patients over age 75 (152). In the ACTIVE W trial, dual antiplatelet therapy with clopidogrel and aspirin was not as effective as warfarin for prevention of systemic embolism, myocardial infarction, stroke, and vascular death (P = 0.0003), with a similar rate of bleeding (153). In the ACTIVE A trial, patients had a decreased rate of thromboembolism when clopidogrel was added to aspirin, however with a significant increase in major bleeding (154). Thus, dual antiplatelet therapy (DAPT) should not be used to prevent stroke in AF patients (124).

The RE-CIRCUIT (Randomized Evaluation of Dabigatran Etexilate Compared to Warfarin in Pulmonary Vein Ablation: Assessment of an Uninterrupted Periprocedural Anticoagulation Strategy) trial revealed that patients who underwent ablation of AF on uninterrupted dabigatran had less major bleeding compared to those who were ablated on uninterrupted warfarin (48, 155). The Venture AF investigators randomized 248 AF ablation patients to uninterrupted rivaroxaban or uninterrupted vitamin K antagonists (VKAs). Adverse events rates were low and similar in both arms of the study. Incidences of major bleeding and thromboembolic events were low (0.4% and 0.8% respectively; 1 major bleeding event, 1 ischemic stroke and 1 vascular death occurred). Each of these events transpired in the VKA arm 1–27 days post-procedure (156). Based on this data, for patients undergoing AF catheter ablation who have been anticoagulated therapeutically for ≥3 weeks with warfarin, rivaroxaban, or dabigatran procedure performance without anticoagulant interruption is guideline recommended (48). Performance of ablation without interruption after therapeutic anticoagulation for ≥3 weeks with apixaban or edoxaban was considered reasonable in the 2017 guidelines (48).

Subsequent studies have examined uninterrupted edoxaban and apixaban vs. uninterrupted VKAs. In the ELIMINATE-AF trial, the incidence of major bleeding was not significantly different between groups. The risk of thromboembolic events was low, two patients suffered strokes (one ischemic and one hemorrhagic), both were in the edoxaban group (157). AXAFA–AFNET 5 compared uninterrupted apixaban to uninterrupted VKAs. The composite primary outcome of death, stroke, or bleeding occurred nearly identically in both groups. The primary apixaban non-inferiority hypothesis was tested using a pre-specified absolute margin of 0.075 (7.5% absolute difference) and verified (non-inferiority *P* = 0.0002). The authors concluded that continuous apixaban is effective and safe for patients undergoing AF ablation (158).

Pre-procedural transesophageal echocardiography performed in patients undergoing AF ablation who have been therapeutically anticoagulated for ≥3 weeks has demonstrated that 1.6–2.1% will have thrombus or “sludge” in the left atrial appendage. Thrombus has been identified in < 0.3% of individuals whose CHA_2_DS_2_-VASc score is zero vs. >5% of those with a CHA_2_DS_2_-VASc score of ≥2. Although clinical practice varies, performing pre-procedural transesophageal echocardiography in all patients presenting for AF ablation regardless of presenting rhythm and anticoagulation status is reasonable. If thrombus is identified in the LAA prior to catheter ablation, the intervention should not be performed (48). Intracardiac echocardiographic (ICE) imaging from the pulmonary artery can be considered for patients who cannot undergo TEE (48).

Once a decision to proceed with AF catheter ablation has been made, heparin should be administered (prior to or immediately after transseptal puncture) and adjusted to achieve and maintain an activated clotting time (ACT) of at least 300 s (48). Unfractionated heparin doses required to achieve this goal are similar for dabigatran and VKAs, but higher heparin doses may be required to achieve this ACT target for ablation on uninterrupted factor Xa inhibitors. It has been speculated that this could result in heparin overdosage and result in increased bleeding (159). Reversing heparin with protamine post-procedure is considered reasonable (48).

Patients who have not been anticoagulated prior to catheter ablation should receive low molecular weight or intravenous heparin as a bridge to initiation of warfarin anticoagulation.

For patients who have not been anticoagulated prior to AF catheter ablation or those whose anticoagulation has been interrupted, it is reasonable to administer a DOAC 3 to 5 h after achieving procedural hemostasis.

Following AF catheter ablation therapeutic anticoagulation with a DOAC or VKAs should be continued for at least 2 months. A decision to continue systemic anticoagulation >2 months after ablation should be based on each patient's stroke risk profile (primarily their CHA_2_DS_2_-VASc score) rather than whether or not the procedure was perceived to be successful. Ideally, discontinuation of anticoagulation based on patient preferences should be accompanied by frequent or continuous ECG monitoring to detect AF recurrence (48).

**Figure 1:**
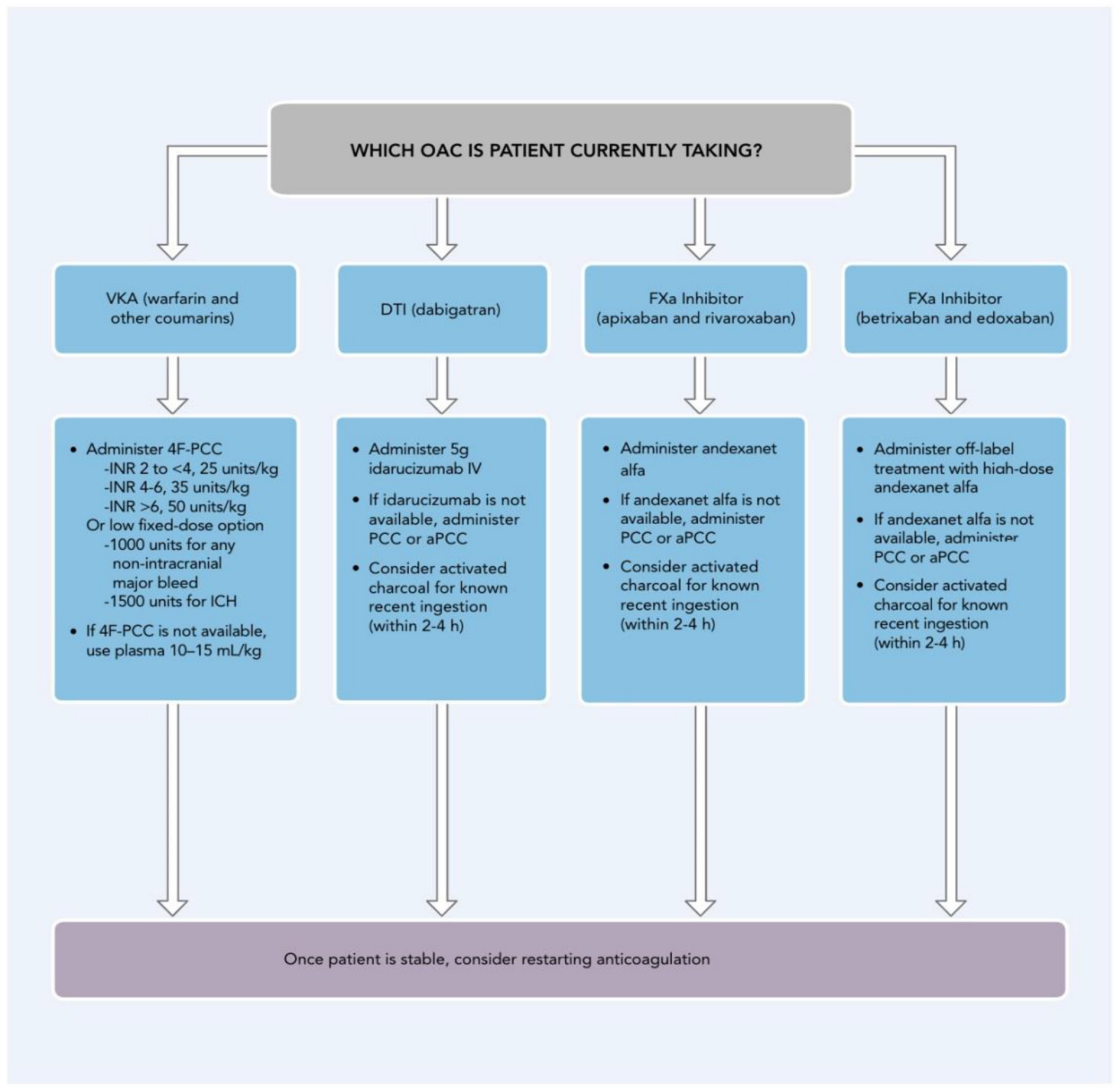
Considerations for Reversal/Hemostatic agents^*^. 4F-PCC, four-factor prothrombin complex concentrate; aPCC, activated prothrombin complex concentrate; DOAC, direct-acting oral anticoagulant; DTI, direct thrombin inhibitor; FXa, Factor Xa; h, hours; ICH, intracranial hemorrhage; INR, international normalized ratio; IV, intravenous; OAC, oral anticoagulant, including DOACs and VKAs; PCC, prothrombin complex concentrate; VKA, vitamin K antagonist; ^*^Reversal/hemostatic agents include repletion strategies such as PCCs, plasma, vitamin K, and specific reversal agents for DOACs (e.g., idarucizumab for dabigatran; andexanet alfa for apixaban or rivaroxaban).^†^When PCCs are used to reverse VKAs, vitamin K should also always be given. Adapted from Tomaselli et al. (160) with permission.

Treatment with VKAs and DOACs may result in major bleeding. A detailed discussion of the management of reversal of coagulopathies related to these agents is beyond the scope of this review. Nevertheless, Tomaselli and associates (160) have suggested a contemporary management approach for oral anticoagulant-induced bleeding which is summarized in Figure 1.

## AF and interventional stroke/thromboembolic prevention

Thrombus in the LAA is the primary source (91%) of thromboembolism in non-rheumatic AF patients (161). The pivotal role of the LAA as a thromboembolic source has made surgical and percutaneous LAA occlusion or excision an important option for patients with contraindications to long term anticoagulation (162). In addition to the patient risk factors previously mentioned (146), poor compliance with or intolerance of anticoagulant therapy are contraindications to long term pharmacologic protection. The implications of a missed dose of a factor Xa or direct thrombin inhibitor are more severe due to their shorter half-lives compared to warfarin and may place the patient at short-term risk for thromboembolism (163). Patients should be taught about dosing and the need for compliance. In order to help prevent adverse drug reactions, patients should be cautioned against initiating therapy with other drugs (prescribed or over the counter) without first speaking to their healthcare provider (164).

In 1999, Man-Son-Hing et al. performed a meta-analysis to assess whether a history of falls should influence prescription of antithrombotic therapy in elderly AF patients. They concluded that a history of or risks factors for falling should not influence the decision to prescribe anticoagulation in elderly AF patients. The risk of fall related subdural hematoma was extremely rare and markedly outweighed by the 6% per year risk of stroke (165). Nevertheless, physician related fear intracranial hemorrhage has continued to limit appropriate anticoagulation (166, 167).

AF patients who have contraindications to long term anticoagulation accompanied by significant risk of thromboembolic complications fall into two categories based on whether (or not) cardiac surgery is planned.

If cardiac surgery is not planned, percutaneous placement of an LAA occlusion (LAAO) device is strongly advised. The currently approved and marketed LAAO devices in the U.S. are the Amplatzer Amulet device (Abbott Vascular, Santa Clara, CA) and the Watchman and Watchman FLX devices (Boston Scientific, Marlborough, MA). The PROTECT AF (WATCHMAN left atrial appendage system for embolic protection in patients with atrial fibrillation) and the PREVAIL (Evaluation of the WATCHMAN LAA closure device in patients with atrial fibrillation vs. long term warfarin therapy) trials randomized non-valvular AF patients (2:1 ratio) to WATCHMAN implantation or warfarin (168). A meta-analysis of these trials' 5-year outcomes demonstrated that, in non-valvular atrial fibrillation, LAA closure with WATCHMAN provided comparable to warfarin stroke prevention, as well as significant reductions in non-procedure-related major bleeding, particularly hemorrhagic stroke, and all-cause mortality (168). The newer-generation WATCHMAN FLX device has improved procedural safety and (in most centers) is replacing the older WATCHMAN device (162).

The WATCHMAN FLX received CE-mark approval in 2015 but was removed from the European market in March 2016 because of increased implant embolization incidents (169).

It is important to note that gender differences may complicate decisions about risks involved in percutaneous LAAO. Using data from the National Cardiovascular Data Registry (NCDR) LAAO Registry, a cohort study of 49,357 patients including 20,388 women (41.3%) and 28,969 (58.7%) men who underwent percutaneous LAAO with the WATCHMAN device was performed. Women were more likely to experience any adverse events (P < 0.001) or major adverse events (due to major bleeding and pericardial effusion requiring drainage) [P < 0.001]. Women were more likely than men to experience a hospital stay >1 day (P < 0.001) and death, albeit minimal, was also more common in women (P < 0.001) (170).

A more recent study, using data on 31,994 patients from the National Cardiovascular Data Registry (NCDR) LAAO Registry, evaluated post-procedure care after LAAO with the WATCHMAN device and compared the risk of adverse events for different antithrombotic discharge strategies in clinical practice. Only 12.2% received the full post-procedure treatment protocol studied in pivotal trials. The most common deviations involved discharge antithrombotic medications. The most frequently prescribed discharge regimens were warfarin and aspirin (36.9%), DOAC and aspirin (20.8%), warfarin only (13.5%), DOAC only (12.3%), and DAPT (5.0%). At 45-day follow-up warfarin alone and DOAC only performed significantly better than the other regimens in frequency of any adverse event. The rate of all adverse events after discharge until the 6-month follow-up was highest in patients receiving warfarin and aspirin (10.3%), followed by dual antiplatelet therapy (9.1%), DOAC and aspirin (9.1%), warfarin (8.5%), and DOAC (8.3%). At 6 months, the major adverse event rates were warfarin and aspirin (9.7%), followed by DAPT (9.3%), DOAC and aspirin (8.9%), warfarin (8.6%), and DOAC (8.0%). Interestingly, at this juncture, only warfarin alone performed significantly better than the other regimens in frequency of adverse events. At both time intervals, differences in adverse events were largely driven by rates of bleeding. No significant differences were found in the risk of stroke or TIA (171).

The PINNACLE FLX study (Protection against embolism for non-valvular AF patients: investigational device evaluation of the watchman FLX LAA closure technology) evaluated the safety and efficacy of the WATCHMAN FLX LAA closure device in NVAF patients who had an appropriate reason (or reasons) to seek a non-pharmacological alternative. Results were reported in 2021 (172).

The primary safety end point was occurrence of one of the following ≤ 7 days post procedure or by hospital discharge, whichever happened later: death, ischemic stroke, systemic embolism, or device/procedure-related complications requiring cardiac surgery. The primary effectiveness end point was the incidence of effective LAA closure (peri-device flow ≤ 5 mm), by echocardiographic assessment at 12-month follow-up (172).

Among the 400 patients enrolled, the mean age was 73.8 ± 8.6 years and their mean CHA_2_DS_2_-VASc score was 4.2 ± 1.5. The 0.5% incidence of the primary safety end point met the performance goal of 4.2% (P < 0.0001). The 100% incidence of the primary effectiveness end point met the performance goal of 97% (P < 0.0001). Device-related thrombus was reported in 7 patients, there were no pericardial effusions that required open cardiac surgery, and (in contrast to the European experience) device embolization did not occur (172).

The Amplatzer Cardiac plug (ACP) 1 (Abbott Vascular, Santa Clara, CA) aimed to seal the body and ostium of the LAA. The mechanism by which the distal lobe and proximal disk for seal the LAA orifice is termed the “pacifier principle” (162, 172). The ACP 1 as well as the Wave Crest device (Biosense Webster, Irvine, CA, USA) are alternative options when the LAA cannot (is too small to) accommodate deeper devices. The Amulet or ACP 2 (Abbott Vascular, Santa Clara, CA) includes significant improvements such as more stabilizing wires, larger disc diameters, longer lobe and waist length. It can be implanted deeper (~12 vs. ~10 mm) inside the LAA cavity and post-deployment adjustment has been facilitated (173, 174).

The recently published Amulet IDE trial (175) randomly (1:1) assigned 1,878 patients with NVAF at increased risk of stroke to the Amplatzer™ Amulet™ (Abbott Vascular, Santa Clara, CA) or the WATCHMAN device. Patients with a CHADS_2_ score ≥ 2 or CHA_2_DS_2−_VASc score of ≥ 3 (averages 4.5 and 4.7 in the Amulet and WATCHMAN groups, respectively) were eligible for enrollment. In addition, patients had to be suitable for 6 months of anticoagulation while having justifiable reason to seek a non-pharmacological alternative (HASBLED averages 3.2 and 3.3 in the Amulet and WATCHMAN groups, respectively).

Implant success rates were similar in the two groups, however unsuitable patient anatomy was less common in the Amulet cohort. Amulet was non-inferior (non-inferiority margin 5.8%, P < 0.001) for the primary composite safety endpoint of all-cause death, or major bleeding through 12 months as well as the primary composite efficacy endpoint of ischemic stroke or systemic embolism (non-inferiority margin 3.2%, P < 0.001) through 18 months. At 45 days, device-based LAA occlusion with a residual jet < 5 mm was significantly better in the Amulet group (P = 0.003) perhaps related to its dual vs. Watchman's single mechanism (173–175).

Procedure-related complications were more common in the Amulet group, possibly as a result of non-European implanters having less experience (175). The Amulet received CE Mark in 2013 (174), while the U.S. Food and Drug Administration (FDA) approved the Amplatzer™ Amulet™ on August 16, 2021, largely as a result of the Amulet IDE trial (176).

For Watchman recipients, 45 days of warfarin followed by 6 months of DAPT after LAA closure is recommended in the Food and Drug Administration–approved label to prevent device related thrombosis (DRT) before the occluder is completely endothelialized (177). DRT rates were similar between in the Amulet IDE trial groups despite the reduced rate of post-procedure anticoagulation in the Amulet group (175). It is currently purported that Amplatzer™ Amulet™ patients may be discharged without oral anticoagulation. In a meta-analysis of observational data from 83 studies including 12,326 patients, there were no differences in the occurrence of stroke, major bleeding, DRT, or all-cause mortality in patients treated with short-term oral anticoagulation or anti-platelet therapy following LAAO. Likewise, there were no differences among patients who received different LAAO devices (178).

The CHAMPION-AF study (WATCHMAN FLX vs. NOAC for Embolic ProtectION in in the Management of Patients With Non-Valvular Atrial Fibrillation) is an FDA-approved randomized controlled trial currently enrolling AF patients in order to compare WATCHMAN FLX to long-term DOAC anticoagulation. It is expected to be completed in 12/27 (162, 179).

The CATALYST trial Clinical Trial of Atrial Fibrillation Patients Comparing Left Atrial Appendage Occlusion Therapy to Non-vitamin K Antagonist Oral Anticoagulants will evaluate the efficacy and safety of the Amplatzer™ Amulet™ device in comparison to DOAC therapy. It is expected to be completed in 4/29 (162, 180).

The LARIAT system requires percutaneous access to cardiac endocardial and the epicardial spaces. An endocardial magnetic guide placed within the LAA to allow an epicardially placed lasso to tie off the LAA (162).

In 2015, the US FDA issued a safety communication stating that death and other complications such as cardiac laceration or perforation or complete LAA detachment from the heart had been associated with LARIAT use (162, 181). To a large extent, this tempered enthusiasm for this approach.

The aMAZE trial (sponsored by AtriCure, Mason, OH, USA) an FDA-approved, prospective, multicenter, randomized controlled, superiority-designed trial evaluated the LARIAT^®^ Suture Delivery Device for LAA exclusion as an adjunctive to PVI catheter ablation for the treatment of persistent and long-standing persistent AF. Its aim was to determine if LAA ligation as adjunct to PVI (vs. PVI alone) increased maintenance of sinus rhythm in patients with persistent and long-standing persistent AF (182, 183). Primary end points included 30-day safety of the LARIAT procedure and freedom from documented AF, atrial flutter, or atrial tachycardia >30 s at 12 months after PVI off antiarrhythmic drugs. Key secondary outcomes included a composite of cardiovascular death and stroke, as well as quality of life. Although the primary safety endpoint was met, left atrial appendage ligation/pulmonary vein antral isolation failed to meet the criterion for effectiveness, since recurrent atrial arrhythmia recurrences were similar between treatment groups (184).

In patients with AF and valvular heart disease for whom surgical intervention is planned, the risks and benefits of simultaneous arrhythmia surgery should be discussed thoroughly.

For symptomatic patients with paroxysmal or persistent AF who are undergoing valve surgery, simultaneous pulmonary vein isolation or a maze procedure can be beneficial to reduce symptoms and prevent arrhythmia recurrence. LA appendage ligation/excision is reasonable to reduce the thromboembolic risk in patients with AF or atrial flutter who are undergoing valve surgery. A role for LAA occlusion has not been established in patients without atrial arrhythmias. In the absence of atrial arrhythmias, LA appendage occlusion/exclusion/amputation is potentially harmful (185).

AF patients with an indication for as well as a concomitant contraindication to long-term anticoagulation, who will be undergoing cardiac surgery for a different indication, are candidates for surgical LAA occlusion. Some experts recommend that AF patients with a CHA_2_DS_2_-VASc score ≥2 undergoing cardiac surgery for a different indication have simultaneous surgical LAA occlusion (162) (see the evidence below). A variety of techniques are available which add little additional morbidity or mortality risk. These include amputation, ligation, stapler closure, or an approved surgical occlusion device such as an AtriClip^TM^ device (AtriCure, Mason, OH, USA) (162, 186, 187). Intraoperative and post-operative TEE confirmation of LAA closure is recommended. After a brief learning curve surgeons achieve nearly 90% success (186).

The LAAOS III Investigators conducted a multicenter, randomized trial involving participants with atrial fibrillation and a CHA_2_DS_2_-VASc score ≥2 scheduled to undergo cardiac surgery for another indication. The mean patient age was 71 and the mean CHA_2_DS_2_-VASc score was 4.2. The study included 2,379 participants in the left atrial occlusion group and 2,391 in the no-occlusion group. At baseline, ~50% of the participants were receiving oral anticoagulation. The assigned procedure was performed in 92.1% of the participants. At 3 years, 76.8% of the participants were receiving oral anticoagulation.

The different risk of stroke between the two groups was more pronounced after the first 30-days post-surgery. The authors postulated that early after surgery, some strokes are probably related to the surgery itself and that after the perioperative period, a larger proportion of strokes are caused by cardiac thromboembolism related to atrial fibrillation, for which occlusion is effective.

Overall, systemic embolism or stroke occurred in 114 participants (4.8%) in the occlusion group and 168 (7.0%) in the no-occlusion group (P = 0.001). The incidence of perioperative bleeding, heart failure, or death was not significantly different between the trial groups.

The authors cautioned that LAAOS III did not compare left atrial appendage occlusion with anticoagulation, and it would be incorrect to conclude that occlusion at the time of surgery should be considered as a replacement for anticoagulation. They concluded that AF patients who had undergone cardiac surgery, most of whom continued to receiving antithrombotic therapy, the risk of stroke or systemic embolism was lower when concomitant left atrial appendage occlusion was performed during the surgery than when it was not (188).

Although implantable loop recorders (ILRs) do not prevent stroke or systemic emboli they play an important diagnostic role in patients at risk of AF as well as for detection of AF as a source of cryptogenic stroke. The diagnostic yield from ILRs (with nearly 3 years of battery life) significantly exceeds 24-h Holter, 30-day event, or 30-day mobile cardiovascular telemetry monitors (189).

## Conclusions

In part 2 of this review, we have discussed AF catheter ablation in heart failure patients, compared the efficacy of various ablation modalities, and provided a view of evolving new techniques (while tempering our vast enthusiasm with words of caution). In addition, we have examined surgical AF ablation, compared catheter and surgical AF ablation, provided an update on hybrid AF ablation, all while focusing on the benefits of early intervention.

To conclude this treatise, we have addressed pharmacological thromboembolic prevention and have provided an overview of peri-procedural management of anticoagulation. Lastly, we have covered the strengths and limitations of percutaneous as well as surgical approaches to preventing AF-related stroke/thromboembolism.

## Author contributions

Study supervision, administrative, technical, or material support, drafting of the manuscript, and study concept and design: RT. Critical revision of the manuscript for important intellectual content: HH and PS. Analysis and interpretation of data, acquisition of data, and full access to all of the data in the study and take responsibility for the integrity of the data and the accuracy of the data analysis: RT, HH, and PS. All authors contributed to the article and approved the submitted version.
